# Multi-modal comparative phenotyping of knock-in mouse models of frontotemporal dementia/amyotrophic lateral sclerosis

**DOI:** 10.1242/dmm.052324

**Published:** 2025-08-26

**Authors:** Sevda Boyanova, Gareth Banks, Tatiana V. Lipina, Rasneer Sonia Bains, Hamish Forrest, Michelle Stewart, Mireia Carcolé, Carmelo Milioto, Adrian M. Isaacs, Sara E. Wells, Frances K. Wiseman

**Affiliations:** ^1^ UK Dementia Research Institute, University College London, London WC1E 6BT, UK; ^2^ UCL Queen Square Institute of Neurology, University College London, London WC1N 3BG, UK; ^3^ School of Science and Technology, Nottingham Trent University, Clifton Lane, Nottingham NG11 8NF, UK; ^4^ University of Toronto, Department of Pharmacology and Toxicology, Toronto Ontario M5G 2C8, Canada; ^5^ Mary Lyon Centre, Medical Research Council Harwell, Oxfordshire OX11 0RD, UK

**Keywords:** Amyotrophic lateral sclerosis, Frontotemporal dementia, Mouse phenotyping

## Abstract

Amyotrophic lateral sclerosis (ALS) and frontotemporal dementia (FTD) are progressive adult-onset neurodegenerative diseases with overlapping pathological and genetic origins. They are caused by multiple underlying mechanisms leading to a common collection of clinical features that occur in a spectrum. Here, we report side-by-side longitudinal behavioural, cognitive and sensory phenotyping of two mouse models of ALS/FTD, to determine which aspects of the disease they recapitulate. We used knock-in models, in which the endogenous mouse orthologues of the *C9orf72* and *TARDBP* (encoding TDP-43) genes have been altered to model specific molecular aspects of ALS/FTD. We found that the *C9orf72^GR400/+^* model exhibits age-related deficit in short-term memory and that parental genotype affects exploration activity in offspring. In the *Tardbp^Q331K/Q331K^* model, we found age-related changes in weight, fat mass, locomotion and marble burying. In both models, we found no evidence of deficits in vision or olfactory habituation-dishabituation. These data provide new insight into genotype-phenotype relationships in these ALS/FTD mice, which can be used to inform model choice and experimental design in future research studies.

## Teaser

**Summary:** We report side-by-side behavioural and sensory phenotyping data of two knock-in next-generation mouse models of aspects of the amyotrophic lateral sclerosis/frontotemporal dementia disease spectrum, which provide insight into genotype-phenotype relationships in these neurodegenerative diseases.

## INTRODUCTION

Frontotemporal dementia (FTD) and amyotrophic lateral sclerosis (ALS) are two progressive neurodegenerative diseases with clinical, neuropathological and genetic overlap (Abramzon et al., 2020; Strong et al., 2017). FTD is a heterogeneous disorder characterised by changes in behaviour, language, executive control and motor symptoms (Olney et al., 2017). In particular, changes in behaviour, such as apathy, loss of empathy and compulsive behaviour, and development of neuropsychiatric symptoms, such as anxiety and depression, are key features of the disease (Schönecker et al., 2024). In later stages of disease, multiple cognitive domains are also impacted, including short- and long-term memory (Poos et al., 2022). The primary symptoms of ALS are associated with motor dysfunction, such as muscle weakness, spasticity and dysphagia. However, up to 50% of patients develop cognitive and/or behavioural impairments during the course of the disease, and 13% of patients show concomitant behavioural variant FTD (Hardiman et al., 2017).

A hexanucleotide GGGGCC repeat expansion in the first intron of the *C9orf72* gene is the most frequent genetic cause of FTD and ALS (collectively C9ALS/FTD), resulting in both loss- and gain-of-function effects (Balendra and Isaacs, 2018; DeJesus-Hernandez et al., 2011; Renton et al., 2011). Three non-mutually exclusive pathogenic mechanisms have been associated with the *C9orf72* hexanucleotide repeat expansion: (1) reduced transcription of the *C9orf72* gene – i.e., loss of function; (2) the accumulation of sense and antisense repeat-containing RNA foci; and (3) expression of toxic dipeptide repeat (DPR) proteins translated from the repeat-containing RNAs mediated by repeat-associated non-ATG initiated (RAN) translation – i.e., gain of function (Balendra and Isaacs, 2018). Functional and genetic analyses suggest that loss of function is not sufficient to drive neurodegeneration by itself (Harms et al., 2013; Jiang et al., 2016; Liu et al., 2016; O'Rourke et al., 2015; Peters et al., 2015; Sudria-Lopez et al., 2016) but can exacerbate gain-of-function effects (Boivin et al., 2020; Jiang et al., 2016). Also, the presence of toxic DPRs is considered a major pathogenic feature of C9ALS/FTD (Milioto et al., 2024). RAN translation occurs in every reading frame and both RNA directions, resulting in five DPRs – polyGA, polyGR, polyGP, polyPA and polyPR, with polyGR, polyPR and polyGA most consistently reported as toxic in *Drosophila* (Mizielinska et al., 2014), mammalian cells (Wen et al., 2014) and mouse models (Choi et al., 2019; Hao et al., 2019; LaClair et al., 2020; Milioto et al., 2024; Verdone et al., 2022; Zhang et al., 2018, 2019).

Importantly, nearly all cases of ALS, half of FTD cases, and most hereditary forms of ALS and FTD are characterised by cytoplasmic mislocalisation and aggregation of the 43 kDa TAR DNA-binding protein (TDP-43) (Neumann et al., 2006). TDP-43 neuropathology is also observed in other neurodegenerative diseases, including approximately half of Alzheimer's disease (AD) cases (Meneses et al., 2021). The pattern of AD-associated TDP-43 neuropathology differs from that seen in ALS/FTD, and the underlying mechanism of its development is unknown. Moreover, ∼50 variants in the gene encoding TDP-43 (*TARDBP*) are causal of ALS and FTD, confirming that TDP-43 plays a mechanistic upstream role in neurodegeneration (Benajiba et al., 2009; Sreedharan et al., 2008).

To further develop mechanistic understanding and test the potential efficacy of new therapies, it is important to understand the clinically relevant phenotypes in animal models. Modelling meaningful behavioural changes in ALS/FTD mice can be challenging because of the effects of disease on emotional domains, which requires holistic phenotyping on a broad range of tasks to maximise translational relevance and value (Ahmed et al., 2017). Previous generation transgenic *C9orf72* and *Tardbp* mouse models show phenotypes that might be partially due to artefacts of different integration sites and/or overexpression of the construct of interest, which may confound the utility of these models for both fundamental and translational research, particularly as *Tardbp* is known to be a dosage-sensitive gene (De Giorgio et al., 2019). To overcome these problems, recently, knock-in (KI) mouse models have been generated for *C9orf72* and *Tardbp* disease-associated mutations.

The novel *C9orf72* KI models show physiological expression of 400 codon-optimised polyGR(GR400) or polyPR(PR400) repeats and a reduction in *C9orf72* gene expression (Milioto et al., 2024). These models have age-dependent spinal motor neuron loss and progressive motor dysfunction on the rotarod with no difference in weight from that of wild-type (WT) littermates. In addition, the GR400 mice show cortical neuronal hyperexcitability (Milioto et al., 2024). The effect of the variant on cognitive, behavioural features relevant to ALS/FTD and sensory function is unknown. The *Tardbp^Q331K/Q331K^* KI model carries a human-equivalent ALS/FTD-associated variant in the endogenous mouse gene, and has been previously reported to exhibit cognitive dysfunction, apathy and perturbed autoregulation of TDP-43 (White et al., 2018).

In this study, we longitudinally investigated the behavioural, cognitive and sensory phenotypes of the novel *C9orf72^GR400/+^* KI model alongside the *Tardbp^Q331K/Q331K^* KI model, to compare the phenotypes of these two next-generation models. We show that *C9orf72^GR400/+^* animals can be used to model not only motor but also cognitive aspects of ALS/FTD, and we confirm that the *Tardbp^Q331K/Q331K^* mouse model recapitulates locomotor deficits and apathy-like symptoms of ALS/FTD, as well as increased weight and increased fat deposition. These data provide critical new insight into genotype-phenotype relationships of aspects of ALS/FTD pathomechanisms, which can be used to inform model choice and experimental design for both fundamental and translational research.

## RESULTS

### Weight and body composition

**Fig. 1.**
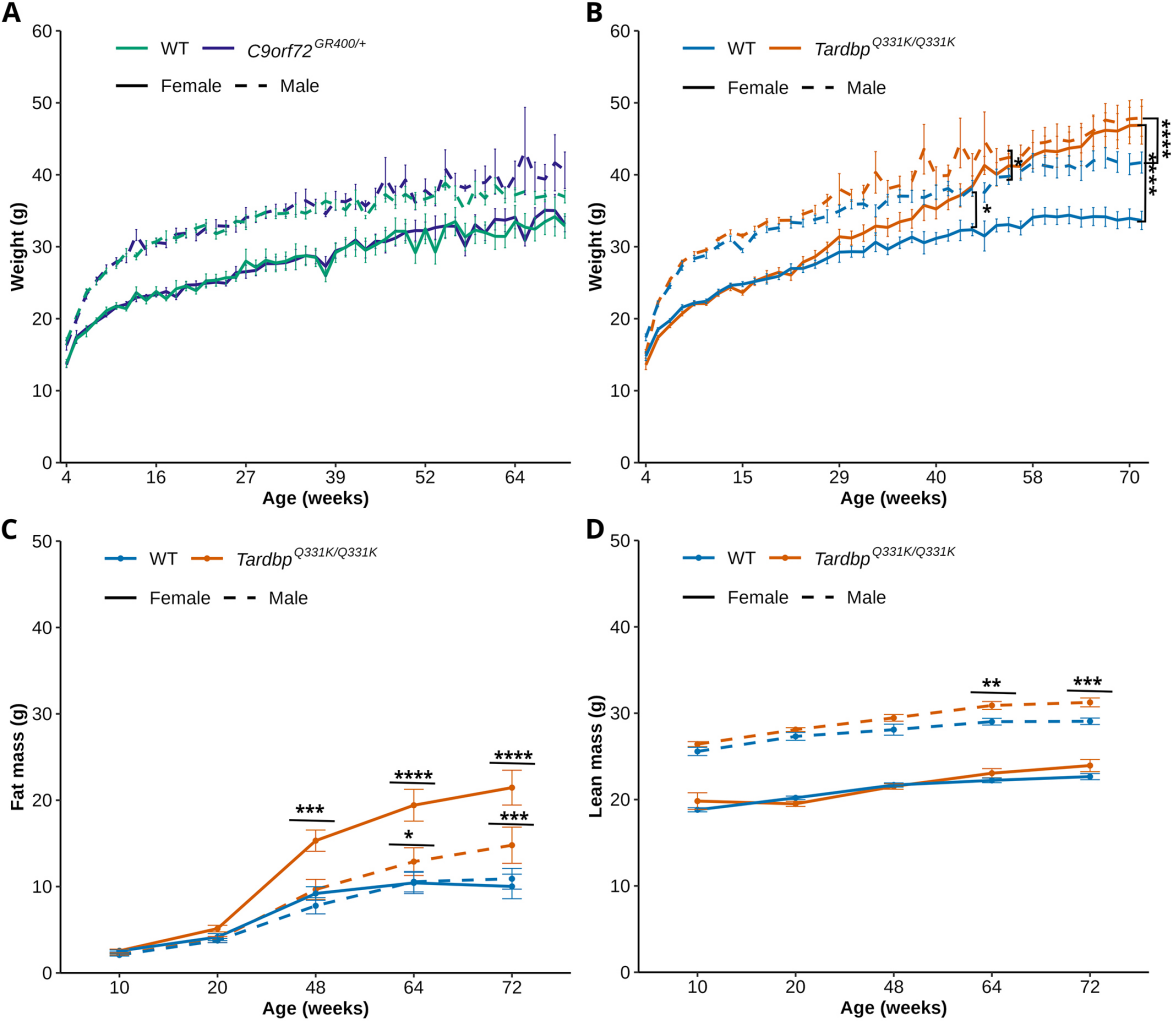
**Weight and body composition in the *C9orf72^GR400/+^* and *Tardbp^Q331K/Q331K^* mouse models.** (A-D) Weight of mice (A,B), fat mass (C) and lean mass (D) measured by echo-MRI was determined in WT female (green solid line), WT male (green dashed line), *C9orf72^GR400/+^* female (purple solid line), *C9orf72^GR400/+^* male (purple dashed line) (A), and WT female (blue solid line), WT male (blue dashed line), *Tardbp^Q331K/Q331K^* female (orange solid line) and *Tardbp^Q331K/Q331K^* male (orange dashed line) (B-D) mice between 4 and 72 weeks of age. (B) Female *Tardbp^Q331K/Q331K^* mice were heavier than female WT mice from 48 weeks of age (*P*=0.0141), and male *Tardbp^Q331K/Q331K^* mice were heavier than male WT mice from 54 weeks (*P*=0.0199). (C) Fat mass was higher in female *Tadbp^Q331K/Q331K^* mice from 48 weeks of age (*P*=0.000212), and higher in male *Tadbp^Q331K/Q331K^* mice from 64 weeks of age (*P*=0.018), compared to that in WT mice of the respective sex. (D) Lean mass was higher in male *Tardbp^Q331K/Q331K^* mice from 64 weeks of age (*P*=0.0046) compared to that in male WT controls. *P<0.05, **P<0.01, ***P<0.001, ****P<0.0001 (linear mixed-effects model and post-hoc analysis with Bonferroni correction). Error bars represent mean±s.e.m. Each point shows an average value±s.e.m for each genotype and sex group; in the weight data, the value for each mouse at each age is an average of one to ten measurements; for echo-MRI, the value for each mouse is from a single measurement. For detailed animal numbers, see Tables S1 and S2. For complete statistical output, see Table S4.

Changes to food intake and metabolism occur in ALS/FTD, with weight loss being a commonly occurring clinical feature of ALS and hyperphagia, and increasing body mass index being associated with behavioural variant FTD (Ahmed et al., 2016a). These changes can confound performance in some motor and behavioural tasks (White et al., 2018). Thus, we determined whether the mouse models exhibited changes in weight from 4 to 72 weeks of age. An age-sex interaction was included in the model because of the known effects of age and sex on mouse weight (Reynolds et al., 2019), and groups were compared by genotype or sex at each age by post-hoc analysis. We found no evidence of an effect of *C9orf72^GR400/+^* genotype on weight (Fig. 1A). However, the expected increase in weight associated with ageing [lmer *F*(50, 1571.27)=265.4233, *P*<2.2×10^−16^] and sex differences in weight [lmer *F*(1, 42,23)=53.1278, *P*=5.457×10^−9^] were observed, such that WT male mice were significantly heavier than females from 8 weeks of age (post-hoc analysis with Bonferroni correction *P*=0.0154), apart from at 57, 68 and 69 weeks of age, and *C9orf72^GR400/+^* males were heavier than females from 8 weeks of age (post-hoc analysis with Bonferroni correction *P*=0.0066), apart from at 68 weeks of age. A significant interaction between the effects of age and sex [lmer *F*(50, 1571.27)=3.0632, *P*=1.107×10^−11^] was also observed, likely because of differences in the development of male and female mice.

We found that female *Tardbp^Q331K/Q331K^* mice were heavier than female WT controls from 48 weeks of age (post-hoc analysis with Bonferroni correction *P*=0.0141) throughout the rest of the study, and male *Tardbp^Q331K/Q331K^* mice were heavier than male WT controls from 54 weeks of age (post-hoc analysis with Bonferroni correction *P*=0.0199) throughout the rest of the study, apart from at 56 weeks of age [genotype effect lmer *F*(1, 50.30)=16.1582, *P*=0.0001952], and a significant interaction between genotype and age [lmer *F*(41, 1674.60)=16.2455, *P*<2.2×10^−16^], (Fig. 1B). In this dataset, we also observed the expected increase in weight associated with ageing [lmer *F*(41, 1674.58)=313.2932, *P*<2.2×10^−16^] and sex differences [lmer *F*(1, 50.29)=36.5988, *P*=1.797×10^−7^], and a significant interaction between age and sex [lmer *F*(41, 1674.59)=3.3519, *P*=9.666×10^−12^]. Longitudinal frailty assessments highlighted no significant welfare concerns in either of the mouse lines used.

To further understand the biology underlying the increased weight in the *Tadbp^Q331K/Q331K^* model, we undertook an echo-magnetic resonance imaging (MRI) study to assess the body composition of mice across the lifespan, including fat and lean mass. Here, we also included an interaction term between age and sex to the statistical model. Fat mass was higher in female *Tadbp^Q331K/Q331K^* mice from 48 weeks of age (post-hoc analysis with Bonferroni correction *P*=0.000212), and higher in male *Tadbp^Q331K/Q331K^* mice from 64 weeks of age (post-hoc analysis with Bonferroni correction *P*=0.018), compared to that in WT controls of the respective sex [main effect for genotype, lmer *F*(1, 52.194)=23.8519, *P*=1.026×10^−5^] (Fig. 1C). We also observed the expected increase in fat mass associated with ageing [lmer *F*(4, 179.274)=185.2816, *P*<2.2×10^−16^] and sex [lmer *F*(1, 52.391)=9.0546, *P*=0.004022], as well as significant interaction between genotype and age [lmer *F*(4, 179.296)=15.4586, *P*=6.982×10^−11^], genotype and sex [lmer *F*(1, 51.913)=5.3641, *P*=0.024539], and age and sex [lmer *F*(4, 179.295)=3.7686, *P*=0.005721]. Lean mass was higher in male *Tadbp^Q331K/Q331K^* mice from 64 weeks of age compared with that in male controls [post-hoc analysis with Bonferroni correction *P*=0.0046; genotype effect lmer *F*(1, 49.137)=11.6177, *P*=0.001313] (Fig. 1D). No difference in lean mass was observed between female *Tadbp^Q331K/Q331K^* mice and female WT controls. We also observed expected effects of age [lmer *F*(4, 178.301)=73.0793, *P*<2.2×10^−16^] and sex [lmer *F*(1, 49.319)=602.5097, *P*<2.2×10^−16^] on lean mass, as well as an interaction between genotype and age [lmer *F*(4, 178.374)=2.9340, *P*=0.022142], although no significant interaction between age and sex was observed. These data indicate that changes in weight in the *Tadbp^Q331K/Q331K^* model are largely the result of increased fat mass, particularly in females, and that progressive changes to lean mass occur in males.

### Sensory function

Olfaction deficits are associated with FTD (Carnemolla et al., 2020); in particular, odour recognition has been reported to be impaired in individuals from the earliest stages of disease. The biology underpinning of this is not well understood, and it has been suggested to be a potentially useful diagnostic tool. Moreover, sensory deficits can impact performance in a range of behavioural tasks in mice (An et al., 2020; De La Zerda et al., 2022; Lipina et al., 2022); in particular, olfaction can impact performance in tests of social behaviour (De La Zerda et al., 2022) and vision tasks with spatial cues (Lipina et al., 2022). Thus, to control for any sensory changes in the mouse models being assessed, we tested olfaction using a habituation and dishabituation task, and visual acuity using an optokinetic drum test.

**Fig. 2.**
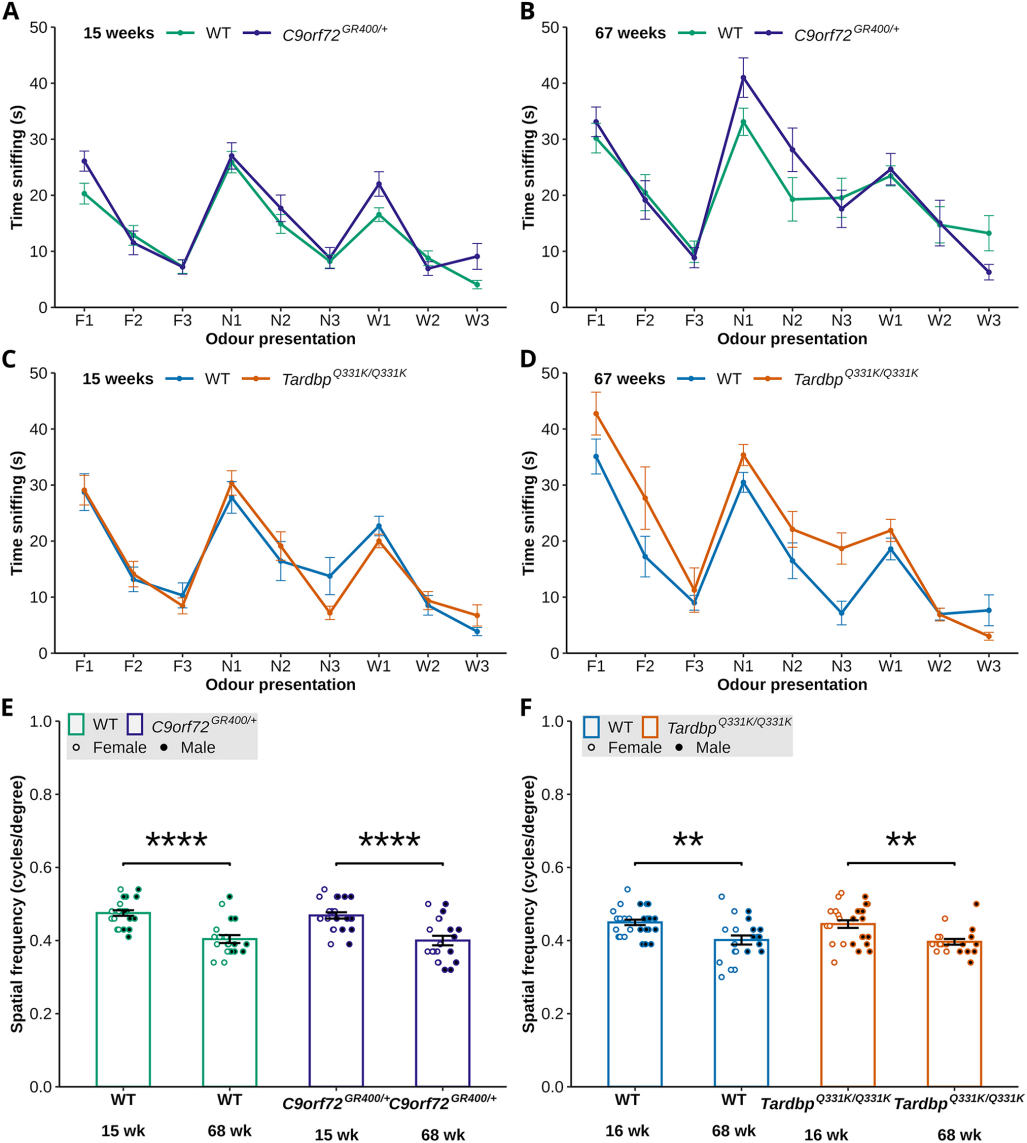
**Assessment of sensory function in the *C9orf72^GR400/+^* and *Tardbp^Q331K/Q331K^* mouse models.** (A-D) Sniffing time, for the first, second and third presentations of familiar social odour (F1, F2, F3), novel social odour (N1, N2, N3) and water odour (W1, W2, W3), to WT (green) and *C9orf72^GR400/+^* (purple) (A,B), and WT (blue) and *Tardbp^Q331K/Q331K^* (orange) (C,D), mice at 15 (A,C) and 67 (B,D) weeks of age. Both models showed normal olfactory habituation and dishabituation. (E,F) Threshold of visual acuity as measured by spatial frequency (cycles/degree) in the optokinetic drum assay at 15-16 and 68 weeks of age in WT (green) and *C9orf72^GR400/+^* (purple) (E), and WT (blue) and *Tardbp^Q331K/Q331K^* (orange) (F), mice. Black circles, males; white circles, females. (E) An age-related decrease in visual acuity in WT (*P*<0.0001) and *C9orf72^GR400/+^* (*P*<0.0001) mice was observed between 15 and 68 weeks of age. (F) An age-related decrease in visual acuity was observed in WT (*P*=0.0012) and *Tardbp^Q331K/Q331K^* (*P*=0.0014) mice between 16 and 68 weeks of age. **P<0.01, ****P<0.0001 (linear mixed-effects model and post-hoc analysis with Bonferroni correction). Error bars represent mean±s.e.m. For olfaction, each point shows average time sniffing±s.e.m.; for each genotype group, each mouse was tested once at every odour presentation. For visual acuity, each circle shows data from a single mouse in one repeat of the test at each age. In the *C9orf72* study, for the olfaction videos, at 15 weeks, WT *n*=24, *C9orf72^GR400/+^ n*=24; at 67 weeks, WT *n*=20, *C9orf72^GR400/+^ n*=18; for visual acuity, at 15 weeks, WT *n*=23, *C9orf72^GR400/+^ n*=23; at 68 weeks, WT *n*=20, *C9orf72^GR400/+^ n*=20. In the *Tardbp* study, for the olfaction videos, at 15 weeks, WT *n*=27, *Tardbp^Q331K/Q331K^ n*=24; at 67 weeks, WT *n*=21, *Tardbp^Q331K/Q331K^ n*=20; for visual acuity, at 16 weeks, WT *n*=27, *Tardbp^Q331K/Q331K^ n*=26; at 68 weeks, WT *n*=21, *Tardbp^Q331K/Q331K^ n*=20. Mice that did not engage with the first presentation of each type of odour were excluded from the olfaction analysis. For complete statistical output, see Table S4.

We used the olfaction task to test the animals' ability to habituate and dishabituate to social odours of familiar or novel mice, and water was used as a control. In *C9orf72^GR400/+^* and WT controls, we observed the expected main effect of odour type and presentation at both 15 weeks of age [lmer *F*(8, 271.407)=36.2347, *P*<2×10^−16^] and 67 weeks of age [lmer *F*(8, 251.857)=18.7708, *P*<2×10^−16^] (Fig. 2A,B). Normal olfactory habituation to social odours and dishabituation from control to social odours was also observed in both genotypes in post-hoc analysis of the time spent sniffing each type of odour (Table S3). In this study, at 15 weeks of age, we also observed an interaction between genotype and parental origin of the mutant allele [lmer *F*(1, 39.181)=5.2138, *P*=0.02791] (Fig. 2A). When this interaction was explored in post-hoc analysis, we observed that *C9orf72^GR400/+^* mice that had inherited their mutant allele from their mother spent more time exploring the first presentation of familiar mouse odour compared to WT controls (*P*=0.0322) (Fig. S3). Because these mice showed a normal pattern of olfactory habituation and dishabituation, we suggest that the observed genotype-parental origin interaction effect could be related to changes in general activity or anxiety, which could be explored further in this model. In *Tardbp^Q331K/Q331K^* mice and WT controls, we observed the expected main effect of odour type and presentation at both 15 weeks of age [lmer *F*(8, 282.464)=30.8712, *P*<2×10^−16^] and 67 weeks of age [lmer *F*(8, 231.165)=27.3237, *P*<2×10^−16^] (Fig. 2C,D). Normal olfactory habituation to social odours and dishabituation from control to social, and between social odours, was observed in both genotypes (Table S2). No main effect of *Tardbp^Q331K^* genotype was observed at either age. These data indicate that neither *C9orf72^GR400/+^* nor *Tardbp^Q331K/Q331K^* mice have significant impairment in olfactory habituation and dishabituation, at the ages tested.

We used a virtual-reality optokinetic drum to test the animals’ visual acuity by measurement of a reflex response. In *C9orf72^GR400/+^* and WT controls, we observed a main effect of age [lmer *F*(1, 40.863)=60.1028, *P*=1.49×10^−9^]; post-hoc analysis with Bonferroni correction demonstrated a reduction in visual acuity in WT (*P*<0.0001) and *C9orf72^GR400/+^* (*P*<0.0001) mice between 15 and 68 weeks of age (Fig. 2E). No effect of *C9orf72^GR400/+^* genotype on visual acuity at either age was detected. In *Tardbp^Q331K/Q331K^* and WT controls, age significantly affected visual acuity [lmer *F*(1, 48.841)=26.9056, *P*=4.09×10^−6^]; post-hoc analysis with Bonferroni correction demonstrated a reduction in visual acuity in WT (*P*=0.0012) and *Tardbp^Q331K/Q331K^* (*P*=0.0014) mice between 16 and 68 weeks of age (Fig. 2F). No effect of *Tardbp^Q331K/Q331K^* genotype on visual acuity at either age was detected. These data indicate that neither *C9orf72^GR400/+^* nor *Tardbp^Q331K/Q331K^* mice have significant impairment in visual acuity at the ages studied; but, in both genotypes, acuity declines with age, consistent with the changes typical of the C57BL6/J genetic background (Banks et al., 2015).

### Behavioural changes

Behavioural changes are a core feature of FTD, in particular apathy, compulsive behaviours, socioemotional deficits and anxiety (Benussi et al., 2021). Recapitulation of these features has previously been reported in a range of FTD mouse models (Ahmed et al., 2017). Here, we determined how the *C9orf72^GR400/+^* and *Tardbp^Q331K/Q331K^* models recapitulate these features, using a range of tasks in a side-by-side comparison.

**Fig. 3.**
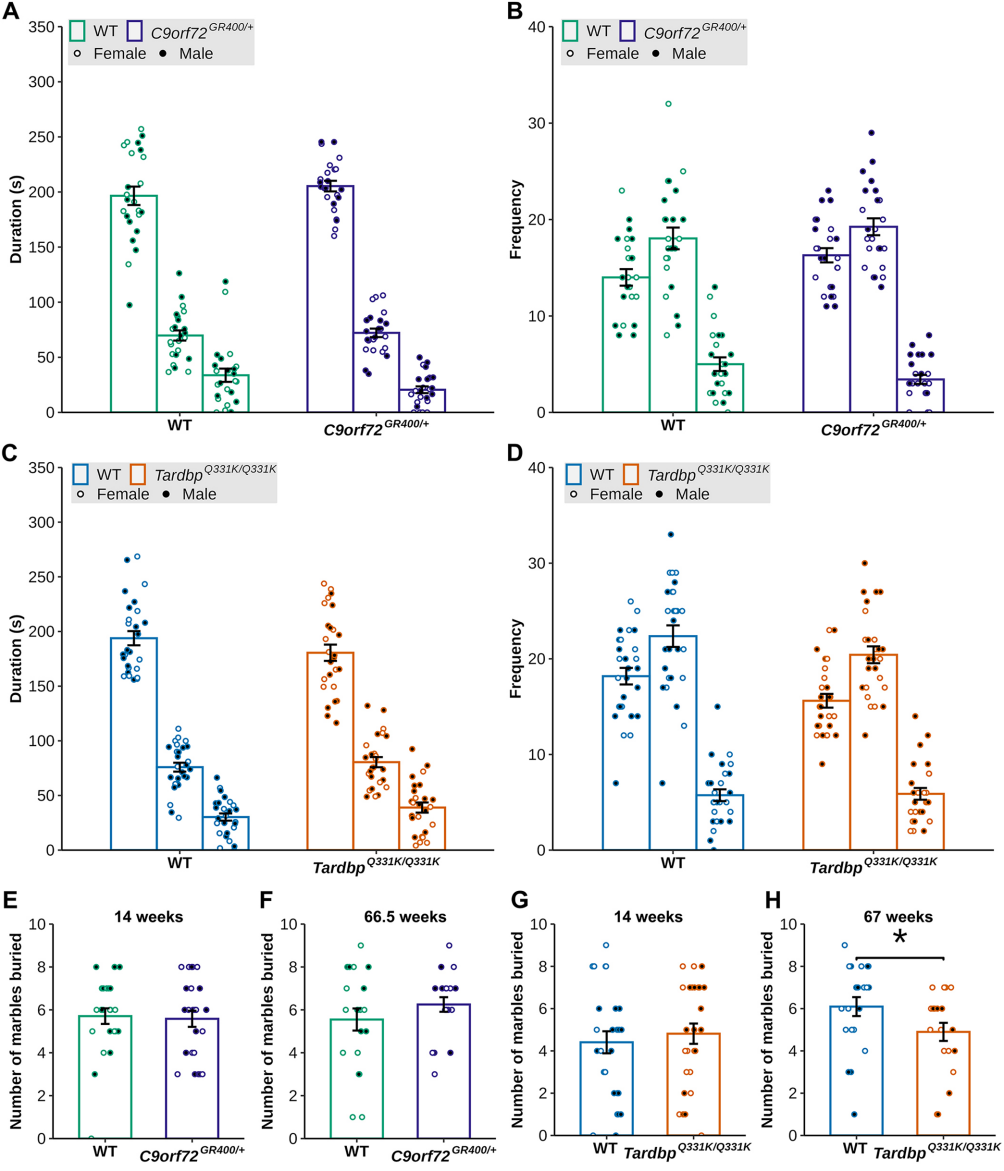
**Assessment of anxiety-like behaviour and general executive functions in the *C9orf72^GR400/+^* and *Tardbp^Q331K/Q331K^* mouse models.** (A-D) Duration and frequency of entry into each section of the elevated plus maze by WT (green) and *C9orf72^GR400/+^* (purple) (A,B), and WT (blue) and *Tardbp^Q331K/Q331K^* (orange) (C,D), mice at 11-12 weeks of age. (E-H) Number of marbles buried in the marble-burying test by WT (green) and *C9orf72^GR400/+^* (purple) mice at 14 and 66.5 weeks (E,F), and WT (blue) and *Tardbp^Q331K/Q331K^* (orange) mice at 14 and 67 weeks (G,H). Black circles, males; white circles, females. The *Tardbp^Q331K/Q331K^* mice buried fewer marbles at 67 weeks of age compared to WT mice (*P*=0.03914). *P<0.05 (Kruskal–Wallis test). Error bars represent mean±s.e.m. Each circle shows data from a single mouse in one repeat of the test at each age, and at each section for the elevated plus maze. In the *C9orf72* study, for elevated plus maze and marble burying at 14 weeks, WT *n*=24, *C9orf72^GR400/+^ n*=24; for marble burying at 66.5 weeks, WT *n*=20, *C9orf72^GR400/+^ n*=20. In the *Tardbp* study, for elevated plus maze, WT *n*=27, *Tardbp^Q331K/Q331K^ n*=26; for marble burying at 14 weeks, WT *n*=27, *Tardbp^Q331K/Q331K^ n*=27; at 67 weeks, WT *n*=21, *Tardbp^Q331K/Q331K^ n*=20. For complete statistical output, see Table S4.

We first investigated anxiety, using the elevated plus maze in naïve animals that had previously not undergone behavioural testing, at 11-12 weeks of age. We compared the time spent and the number of entries into the open sections, closed sections and the centre of the maze. When we assessed the time spent in the different sections of the maze in the *C9orf72^GR400/+^* and WT mice, we found an effect of the section of the maze [lmer(*F*(2, 134)=543.0345), *P*<2×10^−16^] but no effect of genotype, sex or parental origin of the mutant allele. When we analysed the frequency of entries into the different sections of the maze, we observed an effect of the section of the maze [lmer *F*(2, 92)=451.8957, *P*<2.2×10^−16^], and an interaction between genotype and maze section [lmer *F*(2, 92)=7.9614, *P*=0.0006472], but post-hoc comparisons for differences between genotypes within each section did not reach significance (Fig. 3A,B). Thus, in the *C9orf72^GR400/+^* model, we did not find evidence of changes to anxiety using this task. When we assessed the time spent in the different sections of the maze in the *Tardbp^Q331K/Q331K^* and WT mice, we found an effect of section [lmer *F*(2, 151)=438.1583), *P*<2×10^−16^] but no effect of genotype, or sex. When we analysed frequency of entries between sections, we observed an effect of section of the maze [lmer *F*(2, 102)=598.5292, *P*<2×10^−16^], and significant interaction between genotype and section of the maze [lmer *F*(2, 102)=4.6999, *P*=0.01116]; comparison between WT and *Tardbp^Q331K/Q331K^* mice within each section of the maze using post-hoc analysis with Bonferroni correction showed no difference between the genotypes (Fig. 3C,D).

We also assessed marble-burying behaviour in both models at 14 and 66.5-67 weeks of age. This task is considered to represent typical rodent behaviour (De Brouwer et al., 2019) and may result from an impairment in executive function; reduced activity in the task may equate to apathy (Keszycki et al., 2023), increased activity or compulsive behaviour (De Brouwer et al., 2019). No difference in the number of marbles buried was observed in the *C9orf72^GR400/+^* mice compared with controls at 14 weeks of age (Kruskal–Wallis χ²=0.10584, d.f.=1, *P*=0.7449) or at 66.5 weeks of age (Kruskal–Wallis χ²=0.80315, d.f.=1, *P*=0.3702) (Fig. 3E,F). In contrast, an age-dependent reduction in marble burying was observed in *Tardbp^Q331K/Q331K^* mice compared to controls, with no genotype difference in marble burying at 14 weeks of age (Kruskal–Wallis χ²=0.24674, d.f.=1, *P*=0.6194) but a *Tardbp^Q331K/Q331K^*-specific impairment in the task at 67 weeks of age (Kruskal–Wallis χ²=4.2548, d.f.=1, *P*=0.03914) (Fig. 3G,H). These data indicate that the *Tardbp^Q331K/Q331K^* model exhibits an age-related decline in marble burying, which may indicate apathy or reduced locomotor activity in the model. Importantly, a similar phenotype is not seen in the *C9orf72^GR400/+^* model.

**Fig. 4.**
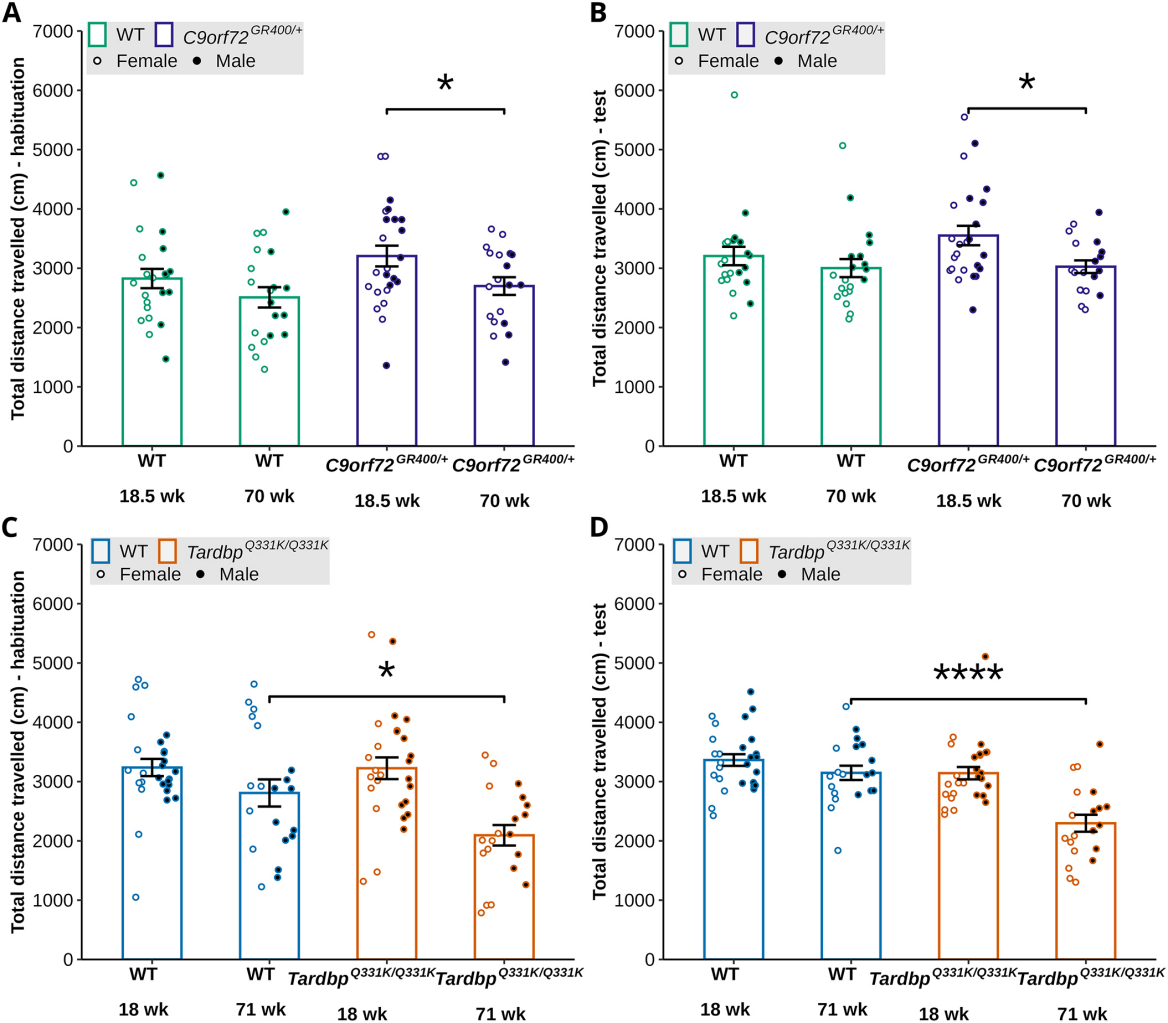
**Locomotor activity during social preference test in the *C9orf72^GR400/+^* and *Tardbp^Q331K/Q331K^* mouse models.** (A-D) Total distance travelled during the habituation and test phases of the social preference test by WT (green) and *C9orf72^GR400/+^* (purple) mice at 18.5 and 70 weeks of age (A,B), and WT (blue) and *Tardbp^Q331K/Q331K^* (orange) mice at 18 and 71 weeks of age (C,D). Black circles, males; white circles, females. (A,B) The 70-week-old *C9orf72^GR400/+^* mice moved less than the 18.5-week-old ones during habituation (*P*=0.0302) (A) and test (*P*=0.0110) (B) phases. (C,D) The *Tardbp^Q331K/Q331K^* mice moved less than WT mice during habituation (*P*=0.0162) (C) and test (*P*<0.0001) (D) phases at 71 weeks of age. *P<0.05, ****P<0.0001 (linear mixed-effects model and post-hoc analysis with Bonferroni correction). Error bars represent mean±s.e.m. Each circle shows data from a single mouse in one repeat of the test at each age. In the *C9orf72* study, at 18.5 weeks, WT *n*=22, *C9orf72^GR400/+^ n*=24; at 70 weeks, WT *n*=20, *C9orf72^GR400/+^ n*=19. In the *Tardbp* study, at 18 weeks, WT *n*=27, *Tardbp^Q331K/Q331K^ n*=27; at 71 weeks, WT *n*=20, *Tardbp^Q331K/Q331K^ n*=20. For complete statistical output, see Table S4.

The Crawley three-chamber social preference test assesses an animal's preference for a social stimulus over a non-social stimulus. Deficits in social preference have been reported to occur in FTD mouse models (Ahmed et al., 2017) and likely relate to the social-emotional changes that occur in FTD. We analysed the models' performances in this task at 18-18.5 and 70-71 weeks of age. We first analysed activity in the task, as determined by the total distance the mouse travelled during the habituation and test phases. In *C9orf72^GR400/+^* mice, we found an effect of age during both parts of the task [habituation lmer *F*(1, 41.058)=7.8548, *P*=0.007701, post-hoc analysis with Bonferroni correction *C9orf72^GR400/+^ P*=0.0302; test lmer *F*(1, 38.859)=9.2378, *P*=0.004227, post-hoc analysis with Bonferroni correction *C9orf72^GR400/+^ P*=0.0110] but no effect of genotype on the total distanced travelled (Fig. 4A,B).

In *Tardbp^Q331K/Q331K^* and WT control mice, we observed a significant effect of age during both parts of the task [lmer habituation *F*(1, 47.656)=29.8108, *P*=1.688×10^−6^; test lmer *F*(1, 42.066)=29.2676, *P*=2.776×10^−6^]. During habituation, we also found an interaction between genotype and age [lmer *F*(1, 47.656)=5.6598, *P*=0.02141], with less activity observed in *Tardbp^Q331K/Q331K^* mice compared to WT controls at 71 weeks of age (post-hoc analysis with Bonferroni correction *P*=0.0162) (Fig. 4C). Similarly, during the test phase of the task, we also found that the total distance travelled was reduced in *Tardbp^Q331K/Q331K^* mice compared with that in WT controls at 71 weeks of age [lmer genotype *F*(1, 46.964)=15.1361, *P*=0.0003141; lmer genotype-age interaction *F*(1, 42.066)=9.2153, *P*=0.0041070; post-hoc analysis with Bonferroni correction *P*<0.0001]. We also found evidence of an effect of sex on the total distance travelled during the test phase [lmer *F*(1, 46.453)=4.5465), *P*=0.0383] (Fig. 4D). These data indicate that a genotype-ageing-related reduction in activity occurs in the *Tardbp^Q331K/Q331K^* model but not in the *C9orf72^GR400/+^* model.

**Fig. 5.**
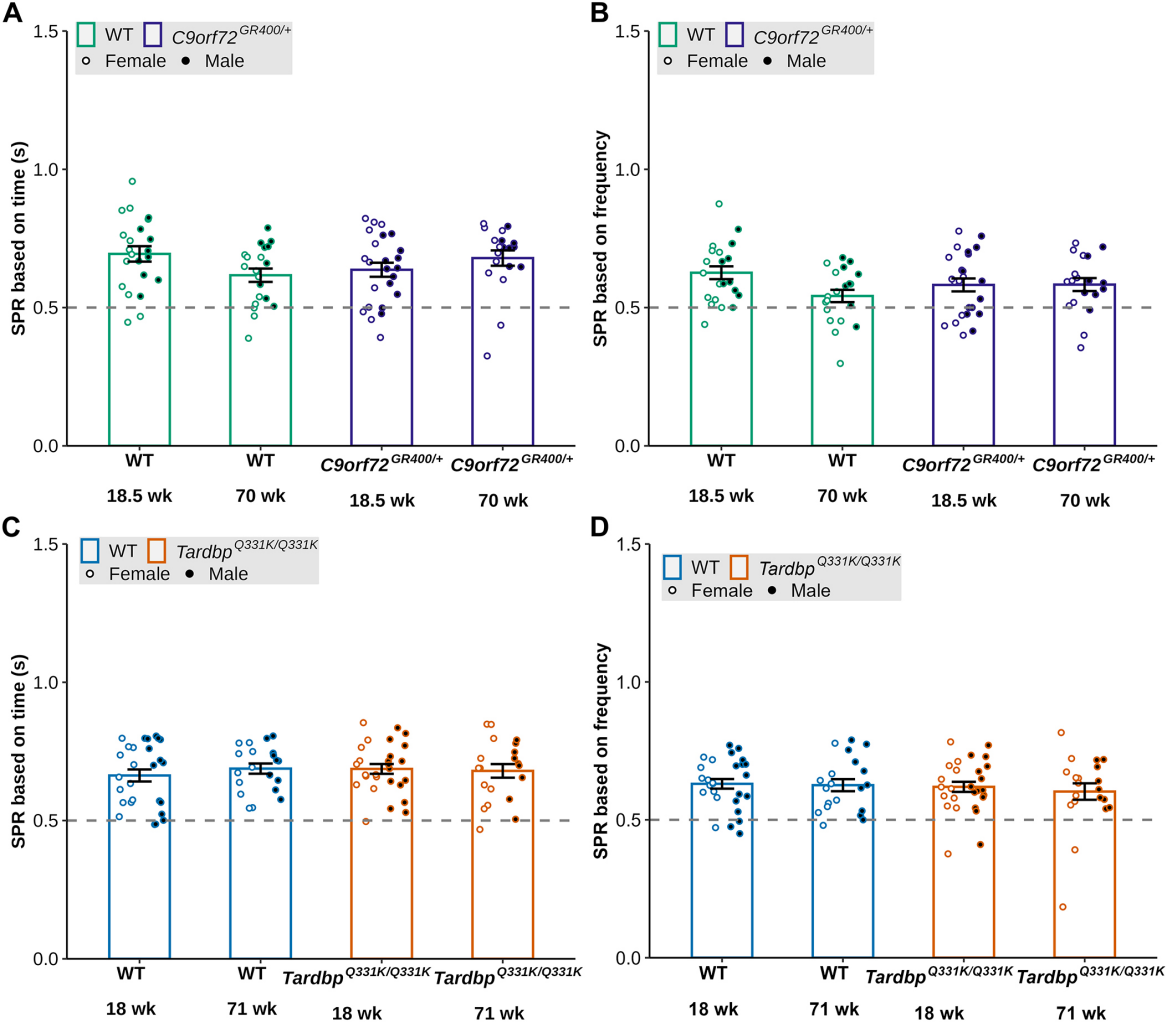
**Social preference ratio (SPR) in the social preference test in the *C9orf72^GR400/+^* and *Tardbp^Q331K/Q331K^* mouse models.** (A-D) SPR based on time (A,C) and frequency (B,D) in WT (green) and *C9orf72^GR400/+^* (purple) mice at 18.5 and 70 weeks (A,B), and WT (blue) and *Tardbp^Q331K/Q331K^* (orange) mice at 18 and 71 weeks (C,D). Black circles, males; white circles, females. *P<0.05, **P<0.01 (linear mixed-effects model and post-hoc analysis with Bonferroni correction). Error bars represent mean±s.e.m. Each circle shows data from a single mouse in one repeat of the test at each age. In the *C9orf72* study, at 18.5 weeks, WT *n*=22, *C9orf72^GR400/+^ n*=24; at 70 weeks, WT *n*=20, *C9orf72^GR400/+^ n*=19. In the *Tardbp* study, at 18 weeks, WT *n*=27, *Tardbp^Q331K/Q331K^ n*=27; at 71 weeks, WT *n*=20, *Tardbp^Q331K/Q331K^ n*=20. For complete statistical output, see Table S4.

We then calculated the social preference ratio based on both the duration and frequency of interactions with the social and non-social stimuli. For social preference determined by duration of interactions, we found significant genotype-age interaction [lmer *F*(1, 77)=5.9038, *P*=0.01744], but no significant post-hoc comparisons were observed between *C9orf72^GR400/+^* and WT controls (Fig. 5A). Similarly, for social preference determined by the frequency of interactions, we saw a significant genotype by age interaction [lmer *F*(1, 77)=4.0001, *P*=0.04902], but post-hoc analysis with Bonferroni correction revealed no significant differences between *C9orf72^GR400/+^* mice and WT controls. (Fig. 5B). For the study of the *Tardbp^Q331K/Q331K^* model, no main effects for social preference determined by time spent or frequency of interactions were detected (Fig. 5C,D). These data indicate that social preference is not altered in either the *Tardbp^Q331K/Q331K^* or *C9orf72^GR400/+^* models at the ages studied, but that performance in this task may decline in the C57BL6/J background during ageing.

To further explore the data from this task, we analysed the time the animals spent exploring the social and non-social stimuli. Here, we studied *C9orf72^GR400/+^* (heterozygous) mice and WT controls, and *Tardbp^Q331K/Q331K^* (homozygous) mice and WT controls. Thus, to generate the phenotyping cohorts for the two lines, different breeding strategies were used. For the *C9orf72^GR400^* study, we bred male or female *C9orf72^GR400/+^* mice with C57BL6/J parents. Thus, whether the male or female parent carries the mutant allele could differently impact the behaviour of the offspring in the phenotyping cohort. For the *Tardbp^Q331K^* study, we bred male and female *Tardbp^Q331K/+^* animals together, such that all matings were genetically identical. Therefore, we could not investigate the effect of the parental origin of the mutant allele in this study.

**Fig. 6.**
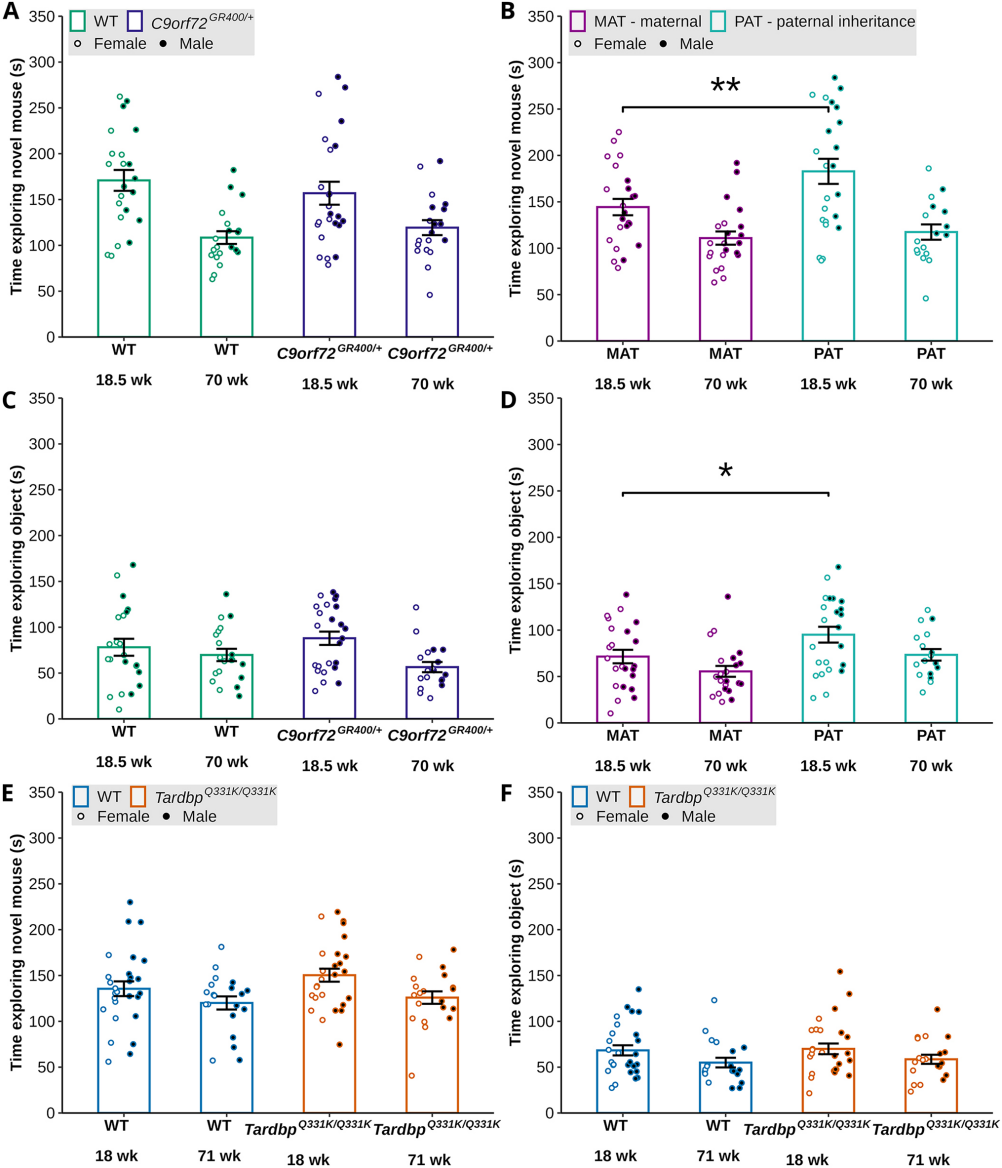
**Time spent with a novel mouse or an object in the social preference test in the *C9orf72^GR400/+^* and *Tardbp^Q331K/Q331K^* mouse models, and the effect of parental mutation inheritance in the *C9orf72^GR400/+^* model.** MAT, maternal; PAT, paternal. Black circles, males; white circles, females. (A-F) Duration spent with a novel mouse (A,B,E) or an object (C,D,F) in the social preference test by WT (green) and *C9orf72^GR400/+^* (purple) mice at 18.5 and 70 weeks (A,C), plotted by genotype irrespective of origin of mutation inheritance. (B,D) Maternal inheritance of the *C9orf72^GR400/+^* mutation (magenta), or paternal inheritance of the *C9orf72^GR400/+^* mutation (turquoise), at 18.5 and 70 weeks, plotted by origin of mutation inheritance, irrespective of genotype. (E,F) Duration spent with a novel mouse (E) or an object (F) in WT (blue) and *Tardbp^Q331K/Q331K^* (orange) mice at 18 and 71 weeks of age. (B,D) In the *C9orf72^GR400^* study, the mice with paternal mutation carrier explored the novel mouse (*P*=0.0093) (B) and the object (*P*=0.0373) (D) longer than the mice with maternal mutation carrier, irrespective of offspring genotype. *P<0.05 (linear mixed-effects model and post-hoc analysis with Bonferroni correction). Error bars represent mean±s.e.m. Each circle shows data from a single mouse in one repeat of the test at each age. In the *C9orf72* study, at 18.5 weeks, WT *n*=22, *C9orf72^GR400/+^ n*=24, MAT *n*=23, PAT *n*=23; at 70 weeks, WT *n*=20, *C9orf72^GR400/+^ n*=19, MAT *n*=22, PAT *n*=17. In the *Tardbp* study, at 18 weeks, WT *n*=27, *Tardbp^Q331K/Q331K^ n*=27; at 71 weeks, WT *n*=20, *Tardbp^Q331K/Q331K^ n*=20. For complete statistical output, see Table S4.

To investigate the potential effect of mode of inheritance (maternal or paternal) in the *C9orf72^GR400/+^* study, we used an age-parental origin of the mutant allele interaction term in our analysis model and split the groups by age and mutant allele origin for post-hoc comparison. Consistent with our analysis of the social preference ratio, we found no effect of *C9orf72^GR400/+^* genotype on the duration of interactions with either stimulus (Fig. 6A,C). However, in this dataset, we observed an effect of age on the duration of interactions with the social stimulus [lmer *F*(1, 39.274)=28.4675, *P*=4.244×10^−6^] and the non-social stimulus [lmer *F*(1, 36.704)=6.9246, *P*=0.012357], and an effect of sex on the duration of interactions with the social stimulus [*F*(1, 38.207)=5.9438, *P*=0.01953]. Interaction between inheritance and sex was included in the model for the time spent with the social stimulus to explore the effect of sex further (Table S4). Interestingly, we also observed an effect from which parent transmitted the *C9orf72^GR400^* allele (maternal/paternal) for both the interaction time with the social [lmer *F*(1, 38.349)=5.4988, *P*=0.02430] and non-social [lmer *F*(1, 35.592)=8.0067, *P*=0.007611] stimuli, such that offspring of *C9orf72^GR400/+^* mothers exhibited reduced exploration at 18.5 weeks of age compared with offspring of *C9orf72^GR400/+^* fathers [post-hoc analysis with Bonferroni correction *P*=0.0093 (social) and *P*=0.0373 (non-social)] (Fig. 6B,D). In the *Tardbp^Q331K/Q331K^* dataset, we found a significant main effect of age on the time spent exploring the social [lmer *F*(1, 88)=6.3402, *P*=0.01361] and non-social [lmer *F*(1, 88)=4.5926, *P*=0.03487] stimuli; however, post-hoc comparisons were not significantly different (Fig. 6E,F). These data indicate that the mode of inheritance of the *C9orf72^GR400^* allele can affect exploration activity in young mice. To determine whether changes in the length of the *C9orf72^GR400^* allele contributed to these differences, we assessed the size of the entire modified allele by touch-down PCR and gel electrophoresis, using DNA extracted from brain tissue of the phenotyped *C9orf72^GR400^* animals (Fig. S1). We found no evidence of a change in size of the allele in any of the samples, indicating that the observed changes in phenotype likely occur independently of any alterations to the number of repeats carried on the mutant allele.

### Test of short-term memory

**Fig. 7.**
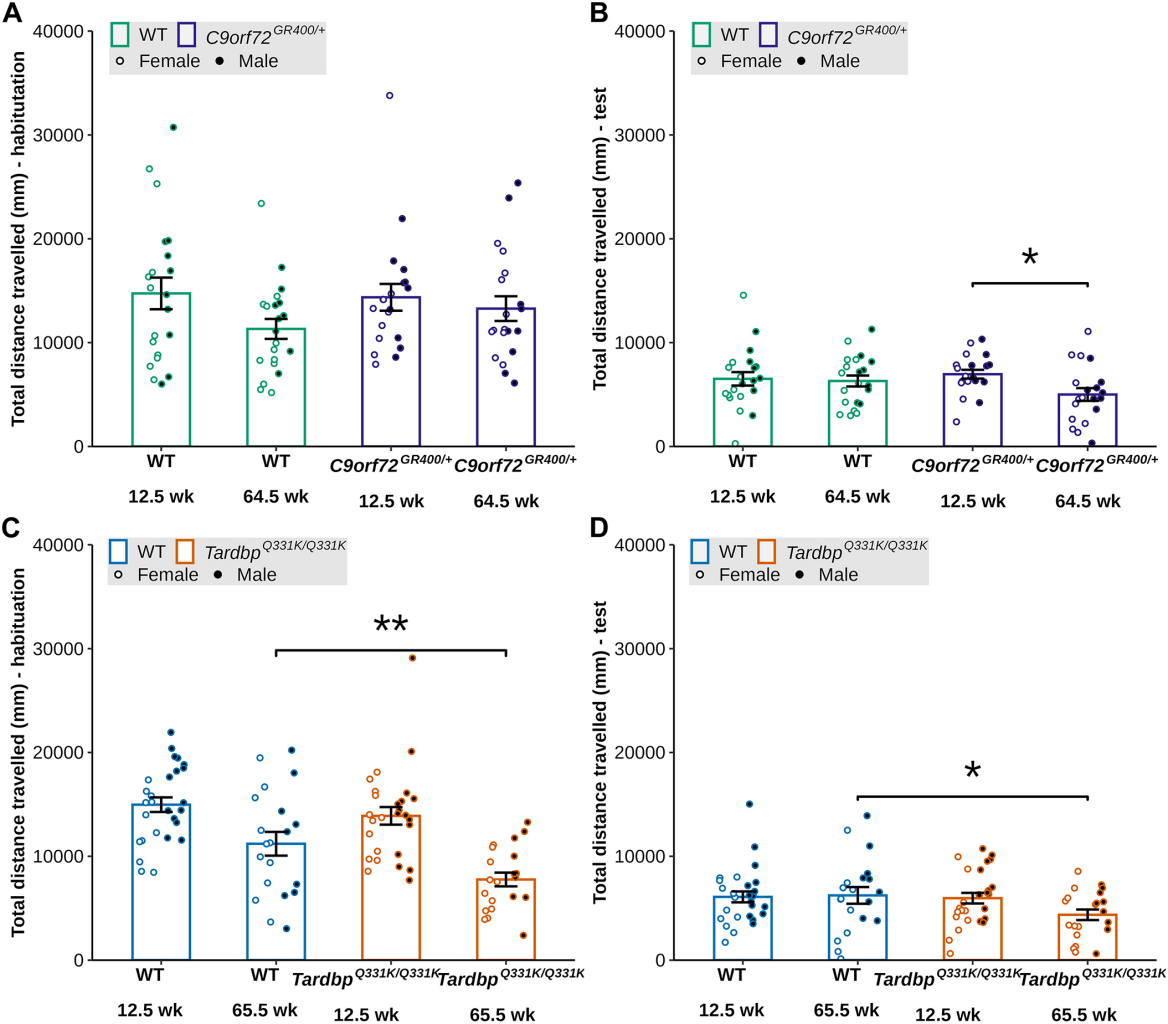
**Locomotor activity during the Y-maze test for short-term spatial memory in the *C9orf72^GR400/+^* and *Tardbp^Q331K/Q331K^* mouse models.** (A-D) Total distance travelled during the habituation (A,C) and test (B,D) phases of the Y-maze test by WT (green) and *C9orf72^GR400/+^* (purple) mice at 12.5 and 64.5 weeks (A,B), and WT (blue), *Tardbp^Q331K/Q331K^* (orange) mice at 12.5 and 65.5 weeks (C,D). Black circles, males; white circles, females. (A) In the *C9orf72^GR400^* study, no significant post-hoc effects were observed for total distance travelled during habituation. (B) The *C9orf72^GR400/+^* mice moved less at the 64.5-week time point than at the 12.5-week time point (*P*=0.0159) during the test phase. (C,D) The *Tradbp^Q331K/Q331K^* mice moved less than WT mice at 65.5 weeks of age, during both habituation (*P*=0.0041) (C) and test (*P*=0.0356) (D) phases. *P<0.05, **P<0.01 (linear mixed-effects model and post-hoc analysis with Bonferroni correction). Error bars represent mean±s.e.m. Each circle shows data from a single mouse in one repeat of the test at each age. In the *C9orf72* study, at 12.5 weeks, WT *n*=21, *C9orf72^GR400/+^ n*=20; at 64.5 weeks, WT *n*=21, *C9orf72^GR400/+  ^n*=20. In the *Tardbp* study, at 12.5 weeks, WT *n*=27, *Tardbp^Q331K/Q331K^ n*=27; at 65.5 weeks, WT *n*=20, *Tardbp^Q331K/Q331K^ n*=21. For complete statistical output, see Table S4.

In addition to the behavioural changes associated with FTD during the later stages of disease, multiple cognitive domains are also affected including short-term memory (Schönecker et al., 2024). To explore whether changes in short-term memory are observed in *C9orf72^GR400/+^* and *Tardbp^Q331K/Q331K^* mice, we tested the animals at 12.5 and 64.5-65.5 weeks of age in a forced alteration spatial Y-maze. We first determined whether the activity was altered during the habituation and test phases of the task by calculating the distance travelled in the maze. In *C9orf72^GR400/+^* and WT mice, we found a main effect of age during habituation [lmer *F*(1, 40.516)=4.8324, *P*=0.0337] and test [lmer *F*(1, 42.926)=5.3118, *P*=0.02608] phases, such that the total distance was reduced in 64.5-week-old *C9orf72^GR400/+^* mice compared with 12.5-week-old animals during the test phase (post-hoc analysis with Bonferroni correction *P*=0.0159), but no difference between genotypes was observed (Fig. 7A,B). In *Tardbp^Q331K/Q331K^* and WT mice, we found a main effect of genotype [lmer *F*(1, 47.779)=6.6680, *P*=0.01294] and age [lmer *F*(1, 43.624)=50.8120, *P*=7.768×10^−9^], and a genotype-age interaction [lmer *F*(1, 43.624)=4.8058, *P*=0.03375], on the total distance travelled in the maze during habituation, such that *Tardbp^Q331K/Q331K^* mice travelled a shorter distance than WT controls at 65.5 weeks of age (post-hoc analysis with Bonferroni correction *P*=0.0041) (Fig. 7C). Similarly, the total distance travelled during the test phase of the task was lower in *Tardbp^Q331K/Q331K^* mice than in WT controls at 65.5 weeks of age [interaction of genotype and age, lmer *F*(1, 43.114)=4.4811, *P*=0.040082; post-hoc analysis with Bonferroni correction *P*=0.0356]. We also observed a main effect of sex [*F*(1, 46.949)=8.6875, *P*=0.004977] (Fig. 7D).

**Fig. 8.**
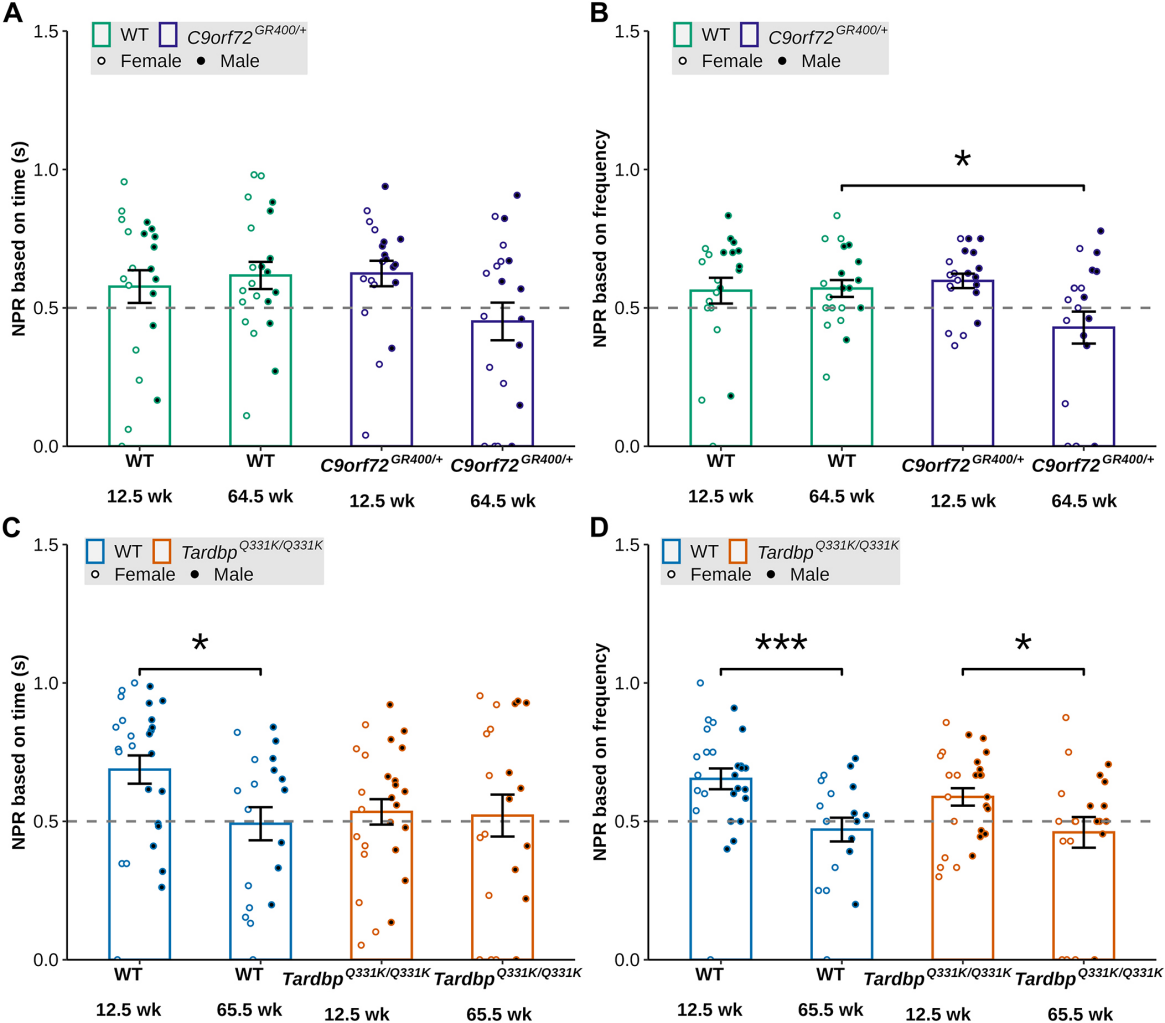
**Novel preference ratio (NPR) in the Y-maze test for short-term spatial memory in the *C9orf72^GR400/+^* and *Tardbp^Q331K/Q331K^* mouse models.** (A-D) NPR based on time (A,C) and frequency (B,D) in WT (green) and *C9orf72^GR400/+^* (purple) mice at 12.5 and 64.5 weeks of age (A,B), and WT (blue) and *Tardbp^Q331K/Q331K^* (orange) mice at 12.5 and 65.5 weeks of age (C,D). Black circles, males; white circles, females. (A) In the *C9orf72^GR400^* study, no significant post-hoc effects were observed when comparing genotypes at each age for NPR based on time (s). (B) The *C9orf72^GR400/+^* model showed genotype-age-related decrease in the NPR based on frequency compared to that in WT mice (*P*=0.0308) at 64.5 weeks of age. (C,D) Age-related decrease in the NPR in the WT (*P*=0.0005) and *Tardbp^Q331K/Q331K^* (*P*=0.0315) mice when calculated based on frequency (D), and only in the WT mice, when calculated based on time (*P*=0.0174) (C). *P<0.05, ***P<0.001 (linear mixed-effects model and post-hoc analysis with Bonferroni correction). Error bars represent mean±s.e.m. Each circle shows data from a single mouse in one repeat of the test at each age. In the *C9orf72* study, at 12.5 weeks, WT *n*=21, *C9orf72^GR400/+^ n*=20; at 64.5 weeks, WT *n*=21, *C9orf72^GR400/+^ n*=20. In the *Tardbp* study, at 12.5 weeks, WT *n*=27, *Tardbp^Q331K/Q331K^ n*=26; at 65.5 weeks, WT *n*=19, *Tardbp^Q331K/Q331K^ n*=21. For complete statistical output, see Table S4.

To investigate whether short-term spatial memory was impaired in the mouse models, we calculated the novelty preference ratio (NPR) for both the amount of time spent in the novel versus familiar arm and the number of entries into each arm. In *C9orf72^GR400/+^* and WT mice, for the NPR (time in arm), we observed a significant interaction between genotype and age [lmer *F*(1, 40.784)=5.4534, *P*=0.02453], but post-hoc analysis did not show significant difference between genotypes at each age (Fig. 8A). In *C9orf72^GR400/+^* and WT mice, for the NPR (frequency of entries), we observed a main effect of sex [lmer *F*(1, 41.318)=5.7870, *P*=0.02070], and a significant interaction between genotype and age [lmer *F*(1, 41.552)=5.6421, *P*=0.02223], such that performance was reduced in *C9orf72^GR400/+^* mice compared to WT controls at 64.5 weeks of age (post-hoc analysis with Bonferroni correction *P*=0.0308) (Fig. 8B). In *Tardbp^Q331K/Q331K^* and WT mice, for the NPR (time in arm), we found no effect of genotype but an effect of age [lmer *F*(1, 44.249)=4.3377, *P*=0.04309], such that in WT mice performance was reduced at 65.5 compared with 12.5 weeks of age (post-hoc analysis with Bonferroni correction *P*=0.0174) (Fig. 8C). Similarly, in *Tardbp^Q331K/Q331K^* and WT mice, for the NPR (frequency of entries), we found no effect of genotype but a main effect of age [lmer *F*(1, 41.727)=21.5518, *P*=3.408×10^−5^], such that across WT (post-hoc analysis with Bonferroni correction *P*=0.0005) and *Tardbp^Q331K/Q331K^* (post-hoc analysis with Bonferroni correction *P*=0.0315) animals, performance was reduced at 65.5 compared with 12.5 weeks of age (Fig. 8D). These data indicate that short-term spatial memory is impaired in the *C9orf72^GR400/+^* mouse model in an ageing-dependent manner, but this phenotype was not detected in the *Tardbp^Q331K/Q331K^* mouse model.

## DISCUSSION

We have performed in-depth, side-by-side behavioural, cognitive and sensory phenotyping of two mouse models of ALS/FTD – the novel model *C9orf72^GR400/+^* and the established model *Tardbp^Q331K/Q331^.* Robust, systematic and consistent comparative phenotyping of these mouse models has allowed us to characterise differences between them and to find specific disease aspects that each model recapitulates. We also determined that the behavioural phenotypes observed in these ALS/FTD models are not confounded by visual or olfactory deficits, as we did not observe any abnormalities in olfactory habituation-dishabituation or in the optokinetic drum test in the lines studied at any age. The ALS/FTD disease spectrum is complex, with numerous genetic and environmental effects reported to influence disease development and progression. Thus currently, no one animal model can encompass all clinical features and/or pathomechanisms (Fisher et al., 2023; Todd and Petrucelli, 2022).

### *C9orf72^GR400/+^* model

In the *C9orf72^GR400/+^* mice, we observed age-related cognitive deficit in the forced alteration Y-maze, suggesting that, as they age, the *C9orf72^GR400/+^* mice show worse performance in this cognitive task of short-term spatial memory, compared to WT mice. Cognitive deficits are an established feature of ALS/FTD, including in *C9orf72* expansion carriers (Chiò et al., 2019; Irwin et al., 2013; Poos et al., 2022); thus, our data indicate that further studies of memory in this model are warranted, to explore how and why memory is impaired in this novel model of ALS/FTD, so as to inform our understanding of this important aspect of ALS/FTD. Moreover, *C9orf72* carriers have reduced attention, even in the asymptomatic stage of the disease (Poos et al., 2022). Thus, in future, studies of attention in this mouse model would be valuable to understand the relationship between attention and memory deficits in disease.

Interestingly, we did not observe genotype effects in the social motivation test in the *C9orf72* study, but we detected a parental inheritance effect. Mice inheriting the *C9orf72^GR400^* mutation from their mother spent a shorter time exploring both the object (non-social) and the novel mouse (social) stimuli. This indicates that a decrease in exploratory behaviour occurs in animals that had mothers that carried the *C9orf72^GR400^* mutation. Alternatively, a direct maternal effect of the *C9orf72^GR400^* mutation on early development may also contribute to this difference in the behaviour of offspring. Currently, little is known about parental mutation carrier effects in C9ALS/FTD. This could be an important area to explore further in human disease and highlights the importance of controlling for parental mutation status in mouse behavioural studies, particularly using the *C9orf72^GR400^* allele.

We did not observe significant *C9orf72^GR400^* allele effects on the total distance moved in the Y-maze and the three chamber-test, confirming that the changes observed in these tasks are not due to effects on general locomotion. This is a particularly important control to perform in mouse models of ALS/FTD, which have motor phenotypes that could affect locomotion and the ability to undertake behavioural tests. Indeed, *C9orf72^GR400/+^* mice show motor deficits in the rotarod from 6 months in males, and from 4 months in females (Milioto et al., 2024).

### *Tardbp^Q331K/Q331K^* model

In the *Tardbp^Q331K/Q331K^* mice, we observed sex and genotype effects on weight, such that mutant animals are heavier than WT littermates, and that this effect is more pronounced in females than in males. Our echo-MRI data on body composition showed that differences in weight correspond with higher fat mass in both females and males, whereas, for lean mass, we observed a significant difference between the genotypes only in male mice. These data reveal differences in the *Tardbp^Q331K/Q331K^* model weight gain between the sexes. Our results are consistent with previous reports of increased weight (Watkins et al., 2021; White et al., 2018) and hyperphagia (White et al., 2018) in this mouse model, as well as previous transgenic systems (Stallings et al., 2013), and extend our understanding of how these phenotypes relate to body composition in male and female mice. Increased body mass index in patients with FTD compared to that in unaffected ageing controls (Ahmed et al., 2016b), and compared to that in AD patients (Ahmed et al., 2019), has been reported. Hyperphagia is also experienced by some people with FTD (Ahmed et al., 2016c), and an increase in total and visceral fat, android:gynoid fat ratio and lean mass is associated with the condition (Ahmed et al., 2019). These changes in body composition have also been correlated with atrophy in complex neuronal networks, including structures involved in reward processing and autonomic function in behavioural variant FTD (Ahmed et al., 2019). Thus, we observe that the *Tardbp^Q331K/Q331K^* model recapitulates changes to body composition that occur in FTD and highlight that this model is useful to understand the underlying systemic and metabolic changes that occur in disease.

In the marble-burying test, burying was reduced in *Tardbp^Q331K/Q331^* mice at 67 weeks of age, consistent with a previous report (White et al., 2018). This may reflect apathy in the model, as has been reported in other tests, for example using the fixed-ratio/progressive-ratio task (Kim et al., 2020). Notably, apathy has been reported in ALS patients who have *TARDBP* mutations (Moglia et al., 2024). Alternatively, the reduced burying phenotype in older *Tardbp^Q331K/Q331K^* mice may reflect the general decrease in locomotion that we and others (Jhuang et al., 2010; White et al., 2018) observe in the model, or may reflect generalised impairment of executive function in the model.

In this study, we did not observe significant *Tardbp^Q331K/Q331K^* genotype effects on short-term spatial memory performance, using the forced Y-maze. In this dataset, we note that the WT group had relatively poor performance at 65.5 weeks of age, which reduced the sensitivity of the task to detect changes in the *Tardbp^Q331K/Q331^* model; because of this limitation, the absence of a phenotype should be interpreted with caution, particularly as previous studies have reported memory decline in 6-month-old *Tardbp^Q331K/Q331K^* mice in the novel object recognition task (3 h delay) (White et al., 2018) and cognitive inflexibility using a visual discrimination and reversal learning task (Ahmed et al., 2016c). Cognitive impairment has been reported in ALS/FTD (Rusina et al., 2021), and cognitive impairment detected by the Edinburgh Cognitive and Behavioural ALS screen has been found to be a valid predictor of TDP-43 pathology in patients with ALS who do not have clinical dementia (Gregory et al., 2020). Thus, further studies of the mechanisms that underlie these ALS/FTD-related changes in memory in preclinical systems will be useful to test the efficacy of new therapies for this key feature of disease.

Here, we observed an ageing effect of the *Tardbp^Q331K/Q331K^* genotype on locomotion in the Y-maze and in the three-chamber task, consistent with a previous report of decreased walking behaviour in the model in male mice at 7.5 months of age (White et al., 2018). This may confound performance in these and other similar tasks, and this feature of the model should be controlled for in studies using the mice. The reduction in locomotion may relate to motor deficits and weight gain in this model (Watkins et al., 2021). Similarly, motor deficits have also been reported in patients with ALS with TDP-43 variants (Kabashi et al., 2008) and are a key clinical feature of disease. The *Tardbp^Q331K/Q331K^* mice robustly model these important aspects of disease and have significant utility for the understanding of underlying mechanisms and efficacy testing of novel therapies.

Here, we used a range of sensory and behavioural tests; however, our methods were not exhaustive, and further testing of additional domains or alternative tests in these models may reveal further deficits. For example, using the three-chamber task, we only tested social motivation and found no evidence of a deficit in either of the models. This task could have been extended to also test the models for changes in social novelty preference and social learning, which may contribute to changes in social behaviour that occur in many people who have FTD (Olney et al., 2017; Schönecker et al., 2024). Similarly, the Y-maze task could have been extended to determine whether long-term memory was altered in the models, as occurs in some people who have FTD (Poos et al., 2022).

Here, we studied two animal models of aspects of FTD/ALS; multiple additional disease mechanisms have been linked to these diseases, including variants in the gene *FUS* (Abramzon et al., 2020), which are associated with early-onset ALS with rapid progression. Moreover, ∼5% of FTD cases exhibit FUS pathological aggregates (Moens et al., 2025). KI gene-targeted *Fus* and next-generation humanised *FUS* transgenic mouse models recapitulate key molecular and cellular changes of ALS/FTD. Further investigation of cognitive function in the subset of these models viable as adults would provide further information on the role of FUS pathology in FTD/ALS cognitive-behavioural changes.

### Conclusions

The results from this study open avenues for further behavioural, metabolic and molecular studies in the two mouse models of features of the ALS/FTD spectrum. Specifically, exploring cognitive phenotypes further in the novel *C9orf72^GR400^* model would be valuable. Particularly, for investigating the relationship between the development of attentional and motivational deficits and changes in memory, tests such as the five-choice serial task (attention) and progressive ratio (motivation) could be used. These could be combined with tests of memory and cognitive flexibility, allowing us to better understand the cognitive deficits in the model, how they relate to equivalent deficits in individuals with ALS/FTD, and underlying molecular and cellular changes. Furthermore, maternal behaviour could be examined further in the novel *C9orf72^GR400/+^* model using home-cage analysis to determine whether this relates to the changes in behaviour we observe in offspring of *C9orf72^GR400/+^* mothers. In the *Tardpbp^Q331K/Q331K^* mice, determining whether these mice recapitulate aspects of metabolic syndrome (Ahmed et al., 2016a) and associated changes in gene expression observed in FTD would pave the way to better understand this important aspect of clinical disease.

As highlighted by our work, currently, no one mouse model recapitulates all aspects of ALS/FTD. Here, our side-by-side standardised sensory and behavioural evaluation demonstrates the phenotypic strengths and limitations of two valuable and complementary mouse models of these diseases, to aid model choice and study design for future mechanistic and preclinical therapy efficacy studies.

## MATERIALS AND METHODS

### Animal welfare and husbandry

All animals were housed and maintained in the Mary Lyon Centre at MRC Harwell under specific pathogen-free conditions, in individually ventilated cages (IVCs) adhering to environmental conditions as outlined in the Home Office Code of Practice. All animal studies were licensed by the Home Office under the Animals (Scientific Procedures) Act 1986 Amendment Regulations 2012 (SI 4 2012/3039), UK, and additionally approved by the Institutional Ethical Review Committees. Mice were randomised, blocked by genotype and sex at the time of weaning, into cages of three to five mice. All mice used in the study were bred in the Mary Lyon Centre at MRC Harwell and were housed in IVCs (Tecniplast BlueLine 1284), on grade 4 aspen wood chips (Datesand, UK), with shredded paper shaving nesting material and small cardboard play tunnels for enrichment. The mice were kept under controlled light (light, 07:00-19:00; dark, 19:00-07:00), temperature (22°C±2°C) and humidity (55%±10%) conditions. They had free access to water (25 ppm chlorine) and were fed *ad libitum* on a commercial diet [SDS Rat and Mouse No. 3 Breeding diet (RM3)]. All procedures and animal studies were carried out in accordance with the Animals (Scientific Procedures) Act 1986, UK, Amendment Regulations 2012 (SI 4 2012/3039).

### Animal genetics

The generation of the *C9orf72^em2.1Aisa^* mice (MGI:6827370), here called the *C9orf72^GR400/+^* mouse model, is described in Milioto et al. (2024). The *C9orf72^GR400/+^* line was maintained on a C57BL/6J background by heterozygote backcross prior to the generation of phenotyping cohorts. Phenotyping cohorts were generated by crossing either male or female *C9orf72^GR400/+^* heterozygotes to C57BL/6J WT mice. We started the study with 48 mice in total: *n*=24 WT [12 females (six with maternal mutation inheritance, six with paternal mutation inheritance), 12 males (six with maternal mutation inheritance, six with paternal mutation inheritance)] and *n*=24 *C9orf72^GR400/+^* (12 females (six with maternal mutation inheritance, six with paternal mutation inheritance), 12 males (six with maternal mutation inheritance, six with paternal mutation inheritance)].

The generation of the *Tardbp^em1Rhbr^* mice (MGI:6157626), here called the *Tardbp^Q331K/Q331K^* mouse model, is described in White et al. (2018). The *Tardbp^Q331K/Q331K^* line was maintained on a C57BL/6J background by heterozygote backcross prior to the generation of phenotyping cohorts. Phenotyping cohorts were generated by heterozygote intercross to generate WT and *Tardbp^Q331K/Q331K^* homozygote animals. The study started with *n*=54 mice in total, *n*=27 WT (12 female, 15 male), *n*=27 *Tardbp^Q331K/Q331K^* (12 female, 15 male).

The WT group for each study – *C9orf72^GR400/+^* and *Tardbp^Q331K/Q331K^* – was from its own respective colony. For mouse numbers across ages in every phenotyping test, split by genotype, sex and parental origin of the mutant allele (where applicable), see Tables S1 and S2.

### Animal genotyping

DNA was extracted from ear biopsy, isolated at postnatal day (P)14 using TaqMan Sample-to-SNP (Applied Biosystems). Mice were genotyped for *C9orf72^GR400^* using TaqMan WT and mutant quantitative PCR assays duplexed with *Dot1l* reference allele. The following primers and probes were used for the *C9orf72^GR400^* mutant allele (forward, 5′-TTCCAGATTACGCTTACCATAC-3′; reverse, 5′-CGACCTCTTCCTCGTCCT-3′) and probe (5′-FAM-TACCTCGTCCACGTCCTCGTCTTC-BHQ1-3′), *C9orf72^+^* WT allele (forward, 5′-CTATTGCAAGCGTTCGGATAATG-3′; reverse, 5′-CTTGGCAACAGCAGGAGAT-3′) and probe (5′-FAM-TGGAATGCAGTGAGACCTGGGATG-BHQ-3′), and reference *Dot1l* allele (forward, 5′-TAGTTGGCATCCTTATGCTTCATC-3′; reverse, 5′-GCCCCAGCACGACCATT-3′) and probe (5′- VIC-CCAGCTCTCAAGTCG-MGBNFQ-3′). Mice were genotyped for the *Tardbp^Q331K^* alleles using allelic discrimination assays, using a common pair of primers for both *Tardbp* alleles (forward, 5′-TCTGCTGGCTGGCTAACAT-3′; reverse, 5′-GGGTGGAGGGATGAACTTTG-3′). To discriminate between the mutant and WT alleles, different probes were used (*Tardbp^Q331K^*, 5′-TET-AACTGCTCTTCAACGCT-BHQ1-3′; *Tardbp^+^*, 5′-FAM-CAACTGCTCTGCAACG-BHQ-3′).

To determine *C9orf72^GR400^* expansion length, DNA was extracted from brain tissue of phenotyped *C9orf72^GR400/+^* animals using a REDExtract-N-Amp™ Tissue PCR Kit (Sigma-Aldrich). The modified region was amplified by touchdown PCR (KOD Xtreme™ Hot Start DNA Polymerase, Sigma-Aldrich), with eight cycles of 74°C, eight cycles of 72°C, eight cycles of 70°C and 35 cycles of 68°C for annealing. The primers used were as follows: forward, 5′-CCCATACGATGTTCCAGATTACGCTTACCC-3′; reverse, 5′-GCAATAAACAATTAGGTGCTATCCAGGCCCAG-3′. Amplicon size was assessed compared to reference controls by gel electrophoresis with 0.5% agarose (Tris-Borate-EDTA) (Fig. S1).

### Experimental design

Group sizes were determined using experimentally determined standard deviations of performance variability in the tests used in C57BL/6J mice at the Mary Lyon Centre when available or from the published literature (Oummadi et al., 2020; Sukoff Rizzo et al., 2018) for power calculations (β=0.80, α=0.05, effect size=15%). Group sizes were then increased by 25% to account for the anticipated ageing-related attrition in C57BL/6J animals. All experimenters were unaware of genotype for data acquisition and analysis.

All behavioural testing was undertaken between 08:00 and 16:00 (light, 07.00:19.00; dark, 19.00:07.00). Both phenotyping cohorts were longitudinally tested in the following order: elevated plus maze (11-12 weeks of age); Y-maze (12-13 weeks of age); marble burying (14-16 weeks of age); olfaction test (14-16 weeks of age); optokinetic drum (15-16 weeks of age); social motivation (14-19 weeks of age); Y-maze (64-66 weeks of age); marble burying (66-67 weeks of age); olfaction test (66-68 weeks of age); optokinetic drum (68 weeks of age); social motivation (69-72 weeks of age). Echo-MRI measures of body composition were performed at 10, 20, 48, 64 and 72 weeks of age (*Tardbp^Q331K^* line only). Body weight measurements were taken at defined time points (week 17, 48 and 64) as part of our study design (Fig. S2). From 48 weeks of age onwards, the mice were weighed weekly to monitor for the development of frailty to enhance welfare. In addition, body weights were also recorded when triggered by a welfare concern; these were considered to be experimentally informative and hence were reported to maximise transparency. Time points with weight measurements of at least *n*=3 mice for both genotypes were included in the body weight analysis, to minimise the impact of individual animal variation and to ensure statistical power for meaningful comparisons across age.

### Frailty assessment

Mouse frailty was assessed using a modified version of the frailty index outlined in Whitehead et al. (2014), for the purposes of enhancing animal welfare. Briefly, mice were scored for the presence of the following parameters: alopecia; body condition; breathing rate/depth; cataracts; coat condition; corneal opacity; dermatitis; diarrhoea; distended abdomen; eye discharge/swelling; gait; kyphosis; loss of fur colour; loss of whiskers; malocclusions; menace reflex; microphthalmia; nasal discharge; penile/vaginal prolapse; piloerection; rectal prolapse; righting reflex; tail stiffening; tremor; tumours; vestibular disturbance; vision loss (visual placing). A total frailty score was then taken as the sum of individual parameter scores.

### Elevated plus maze

The mice were transported in their home cages to the test room and allowed to acclimatise for 30 min. For the test, each mouse was taken out of its home cage and positioned in the centre of the elevated plus maze (height from the ground, 47.5 cm; height of the walls of the closed sections, 20 cm; width of the arms, 5 cm; length of the arms, 66 cm). The mouse was allowed to explore the maze freely for 5 min. The experimental trial was video recorded and tracked live using an Ethovision XT15 (Noldus, The Netherlands). The duration (seconds) and frequency of entries in the open sections, closed sections and the centre were analysed.

### Marble burying

Marble burying in mice is used to assess apathy-like behaviour and normal daily behaviours (Angoa-Pérez et al., 2013; Deacon, 2006). An IVC (height, 14 cm; width, 19 cm; length, 35 cm) was filled with sawdust bedding to a depth of ∼4 cm, and nine marbles were placed on the top of the bedding. A single animal was placed into the cage, and the IVC top (without the wire lid) was placed on top. The mouse was left undisturbed for 30 min, before it was removed from the test cage and returned to the home cage. The number of buried marbles (three-quarters or more covered in sawdust) was then recorded.

### Forced-alteration Y-maze

Spatial novelty preference as a short-term memory measure, was assessed in a Y-maze as described previously (Sanderson et al., 2009). Briefly, we used a Perspex^®^ Y-maze with arms at 120° (arm dimensions: height, 20 cm; width, 8 cm; length, 30 cm) with three visual cues at the end of each arm: white plus sign on black background, black circle on white background, and black and white stripes. The mice were assigned two arms (the ‘start’ and the ‘familiar’ arm) to which they were exposed during the first phase (the habituation phase), for 5 min. Timing of the 5 min period began only once the mouse had left the start arm. The mouse was then removed from the maze and returned to its home cage for a 1 min interval between the habituation and the test phases. During the test phase, mice were allowed free access to all three arms. Mice were placed at the end of the start arm and allowed to explore all three arms for 2 min from when they entered the start arm. An entry into an arm was defined by a mouse placing all four paws inside the arm. Similarly, a mouse was considered to have left an arm if all four paws were placed outside the arm. The amount of time spent in each arm, the number of entries made into each arm and the total distance moved were recorded using the Noldus Ethovision XT15. An NPR was calculated based on the time spent or the number of entries into the arms: NPR=novel arm/(novel+familiar arm). Mice that did not leave the start arm during the test phase were not included in the analysis.

### Optokinetic drum

Visual acuity was assessed by head tracking response to a virtual-reality optokinetic system (Stoelting Co., USA) as described by Douglas et al. (2005). Briefly, each mouse was placed onto a podium in an area comprising computer monitors as walls and a mirrored floor. The animal was monitored by a camera built into the lid of the arena. A vertical sine wave rotates around the monitors, and the head and neck movements of the mouse are used to assess how well the mouse tracks the sine wave rotation. The spatial frequency of the lines was increased until there was no longer a response from the animal, indicating that the stimulus is no longer perceived. Grating is measured in cycles per degree.

### Social preference (three-chamber test)

The mice were allowed to acclimatise to the test room for at least 30 min prior to the start of the experiment. The test was carried out in an arena separated into three chambers separated with doors (overall outside dimensions of the apparatus: height, 25 cm; width, 39.1 cm; length, 58.5 cm; inside dimensions of each chamber: height, 25 cm; width, 37.9 cm; length, 18.6 cm). In the first phase of the experiment – habituation, the doors of both chambers were open, and the mouse was placed in the central chamber and allowed to explore freely the entire arena for 10 min. In the next phase of the experiment – test, both sides of the chamber had either an empty weighted wire cage or a weighted wire cage with a novel mouse. The doors of both chambers were open, and the mouse was placed in the central chamber and allowed to explore freely the entire arena. The time and frequency of entries into each of the side chambers (with a novel mouse or with an object), as well as the total distance moved, was recorded using video recording and the Noldus Ethovision XT15. Apart from the time, frequency and distance travelled, we analysed the social preference ratio, calculated as time/frequency spent with the novel mouse divided by the time or frequency with the novel mouse plus the object. The allocation of novel mouse was counterbalanced for genotype and sex, and interactions were annotated manually by an experimenter unaware of genotype.

### Olfaction test

Mice were removed from their home cage and allowed to acclimatise to a clean IVC placed in a home cage analysis rig (Actual Analytics Ltd, Edinburgh, UK) for 30 min prior to the start of the test without access to food but access to water. The odours used for this test were water (control), familiar mouse and novel mouse (social odours) presented on sterile cotton swabs through the access for the water bottle of the IVC. The cotton swabs for water were prepared by pipetting 50 µl deionised water onto the swab. The social odours were prepared by wiping the cotton swab in a zigzag fashion across the bottom of a used cage, either the animal's home cage or an unfamiliar cage. After 30 min, the water bottle was removed, and the first cotton swab was presented in the cage through the water bottle access with the food hopper in place. The mouse was allowed to explore the cotton swab freely for 2 min, followed by a 1 min inter-trial interval. Each odour type was presented three times in a row. The order of the odour types was counterbalanced and randomised across the mouse cohorts.

The time spent sniffing was scored manually using SimpleVideoCoder (Barto et al., 2017); the scorer was unaware of the mouse genotype. We scored the time the mouse spent sniffing each cotton bud. Sniffing was defined as orientation of the mouse's head and nose towards the cotton bud, and distance of the nose at least 1 cm away from the front and the bottom of the food hopper, as well as the lower one-third of the back of the food hopper. Licking or biting of the cotton bud was not included in the time scored. If the mouse spent less than 10 s interacting with the first presentation of a given odour, this run and the consecutive two presentations of the same odour were not used in the analysis, because the mouse failed to sufficiently engage with the stimulus on the first presentation (olfactory habituation and dishabituation, Stanford Behavioral and Functional Neuroscience Laboratory).

### Echo-MRI

WT and *Tardbp^Q331K/Q331K^* littermates were assessed for whole-body content of lean mass, fat mass, total water and free water using echo-MRI (EMR-136-M, EchoMRI, Houston, TX, USA) as per the manufacturer's instructions.

### Statistical analysis

The linear mixed-effects models (lmer) function from the lme4 and lmerTest packages (Bates et al., 2015; Kuznetsova et al., 2017) in R Studio (R version 4.4.2) was used for data analysis, based on Spires-Jones Laboratory (2021). We applied the model: dependent variable∼genotype*(age+parental origin of *C9orf72^GR400^* allele+sex) for the *C9orf72^GR400^* study, or dependent variable∼genotype*(age+sex) for the *Tardbp^Q331K^* study, with the individual animal as a random effect, unless otherwise specified. In cases in which the lmer model showed singularity for the random effect, we ran the model without the random factor using the linear model (lm) function. When there was no difference in conclusions between the lm and lmer results, we report the results from the lmer for consistency throughout the paper. Factor significance was assessed using type III ANOVA and is reported. We performed analysis of the effect of age using the average age of the mice at every time point; thus, we had two age groups for each model. For the olfaction test, the age factor was replaced by odour to confirm the functionality of the test at each time point. Odour was defined as all three presentations of each odour – water, familiar mouse and novel mouse odour, and the two age groups were analysed separately for each mouse model. In the elevated plus maze, which was performed only at one age, the age factor was replaced with section of the maze. The emmeans package with Bonferroni correction was used for post-hoc analysis of comparisons of interest when a significant main effect of a variable was observed to identify which groups differed. We note that this conservative statistical approach leads to an elevated risk of type II error. For count data in the marble-burying test, we used the Kruskal–Wallis test. The significance value was set at *P*<0.05 for all statistical tests. Mice were not used in the analysis if they were culled for welfare reasons, or if there was a procedural failure during the test, corrupted data or video file, or non-engagement with the task. For full details, see Tables S1 and S2. Code and data are available at https://github.com/sboya23/C9orf72-Tdp43-behavioural-analysis. ChatGPT was used to optimise and debug R code.

## Supplementary Material

10.1242/dmm.052324_sup1 Supplementary information

## References

[DMM052324C1] Abramzon, Y. A., Fratta, P., Traynor, B. J. and Chia, R. (2020). The overlapping genetics of amyotrophic lateral sclerosis and frontotemporal dementia. *Front. Neurosci.* 14, 42. 10.3389/fnins.2020.00042 32116499 PMC7012787

[DMM052324C2] Ahmed, R. M., Irish, M., Piguet, O., Halliday, G. M., Ittner, L. M., Farooqi, S., Hodges, J. R. and Kiernan, M. C. (2016a). Amyotrophic lateral sclerosis and frontotemporal dementia: distinct and overlapping changes in eating behaviour and metabolism. *Lancet Neurol.* 15, 332-342. 10.1016/S1474-4422(15)00380-4 26822748

[DMM052324C3] Ahmed, R. M., Irish, M., Henning, E., Dermody, N., Bartley, L., Kiernan, M. C., Piguet, O., Farooqi, S. and Hodges, J. R. (2016b). Assessment of eating behavior disturbance and associated neural networks in frontotemporal dementia. *JAMA Neurol.* 73, 282. 10.1001/jamaneurol.2015.4478 26810632

[DMM052324C4] Ahmed, R. M., Caga, J., Devenney, E., Hsieh, S., Bartley, L., Highton-Williamson, E., Ramsey, E., Zoing, M., Halliday, G. M., Piguet, O. et al. (2016c). Cognition and eating behavior in amyotrophic lateral sclerosis: effect on survival. *J. Neurol.* 263, 1593-1603. 10.1007/s00415-016-8168-2 27260291

[DMM052324C5] Ahmed, R. M., Irish, M., Van Eersel, J., Ittner, A., Ke, Y. D., Volkerling, A., Van Der Hoven, J., Tanaka, K., Karl, T., Kassiou, M. et al. (2017). Mouse models of frontotemporal dementia: a comparison of phenotypes with clinical symptomatology. *Neurosci. Biobehav. Rev.* 74, 126-138. 10.1016/j.neubiorev.2017.01.004 28088537

[DMM052324C6] Ahmed, R. M., Landin–Romero, R., Liang, C. T., Keogh, J. M., Henning, E., Strikwerda–Brown, C., Devenney, E. M., Hodges, J. R., Kiernan, M. C., Farooqi, I. S. et al. (2019). Neural networks associated with body composition in frontotemporal dementia. *Ann. Clin. Transl. Neurol.* 6, 1707-1717. 10.1002/acn3.50869 31461580 PMC6764740

[DMM052324C7] An, Y., Guan, X., Ni, Y., Zhao, Y., Chen, Z., Chen, Y. and Zhang, J. (2020). Reversible olfactory dysfunction impaired learning and memory with impaired hippocampal synaptic plasticity and increased corticosterone release in mice. *Neurochem. Int.* 138, 104774. 10.1016/j.neuint.2020.104774 32474176

[DMM052324C8] Angoa-Pérez, M., Kane, M. J., Briggs, D. I., Francescutti, D. M. and Kuhn, D. M. (2013). Marble burying and nestlet shredding as tests of repetitive, compulsive-like behaviors in mice. *J. Vis. Exp.* 82, 50978. 10.3791/50978 PMC4108161 24429507

[DMM052324C9] Balendra, R. and Isaacs, A. M. (2018). C9orf72-mediated ALS and FTD: multiple pathways to disease. *Nat. Rev. Neurol.* 14, 544-558. 10.1038/s41582-018-0047-2 30120348 PMC6417666

[DMM052324C10] Banks, G., Heise, I., Starbuck, B., Osborne, T., Wisby, L., Potter, P., Jackson, I. J., Foster, R. G., Peirson, S. N. and Nolan, P. M. (2015). Genetic background influences age-related decline in visual and nonvisual retinal responses, circadian rhythms, and sleep. *Neurobiol. Aging* 36, 380-393. 10.1016/j.neurobiolaging.2014.07.040 25179226 PMC4270439

[DMM052324C11] Barto, D., Bird, C. W., Hamilton, D. A. and Fink, B. C. (2017). The simple video coder: a free tool for efficiently coding social video data. *Behav. Res.* 49, 1563-1568. 10.3758/s13428-016-0787-0 PMC5298951 27503301

[DMM052324C12] Bates, D., Mächler, M., Bolker, B. and Walker, S. (2015). Fitting linear mixed-effects models using lme4. *J. Stat. Soft.* 67, 1-48. 10.18637/jss.v067.i01

[DMM052324C13] Benajiba, L., Le Ber, I., Camuzat, A., Lacoste, M., Thomas–Anterion, C., Couratier, P., Legallic, S., Salachas, F., Hannequin, D., Decousus, M. et al. (2009). *TARDBP* mutations in motoneuron disease with frontotemporal lobar degeneration. *Ann. Neurol.* 65, 470-473. 10.1002/ana.21612 19350673

[DMM052324C14] Benussi, A., Premi, E., Gazzina, S., Brattini, C., Bonomi, E., Alberici, A., Jiskoot, L., Van Swieten, J. C., Sanchez-Valle, R., Moreno, F. et al. (2021). Progression of behavioral disturbances and neuropsychiatric symptoms in patients with genetic frontotemporal dementia. *JAMA Netw. Open* 4, e2030194. 10.1001/jamanetworkopen.2020.30194 33404617 PMC7788468

[DMM052324C15] Boivin, M., Pfister, V., Gaucherot, A., Ruffenach, F., Negroni, L., Sellier, C. and Charlet–Berguerand, N. (2020). Reduced autophagy upon C9ORF72 loss synergizes with dipeptide repeat protein toxicity in G4C2 repeat expansion disorders. *EMBO J.* 39, e100574. 10.15252/embj.2018100574 31930538 PMC7024836

[DMM052324C16] Carnemolla, S. E., Hsieh, J. W., Sipione, R., Landis, B. N., Kumfor, F., Piguet, O. and Manuel, A. L. (2020). Olfactory dysfunction in frontotemporal dementia and psychiatric disorders: a systematic review. *Neurosci. Biobehav. Rev.* 118, 588-611. 10.1016/j.neubiorev.2020.08.002 32818582

[DMM052324C17] Chiò, A., Moglia, C., Canosa, A., Manera, U., Vasta, R., Brunetti, M., Barberis, M., Corrado, L., D'Alfonso, S., Bersano, E. et al. (2019). Cognitive impairment across ALS clinical stages in a population-based cohort. *Neurology* 93, e984-e994. 10.1212/WNL.0000000000008063 31409738 PMC6745732

[DMM052324C18] Choi, S. Y., Lopez-Gonzalez, R., Krishnan, G., Phillips, H. L., Li, A. N., Seeley, W. W., Yao, W.-D., Almeida, S. and Gao, F.-B. (2019). C9ORF72-ALS/FTD-associated poly(GR) binds Atp5a1 and compromises mitochondrial function in vivo. *Nat. Neurosci.* 22, 851-862. 10.1038/s41593-019-0397-0 31086314 PMC6800116

[DMM052324C19] De Brouwer, G., Fick, A., Harvey, B. H. and Wolmarans, D. W. (2019). A critical inquiry into marble-burying as a preclinical screening paradigm of relevance for anxiety and obsessive–compulsive disorder: mapping the way forward. *Cogn. Affect. Behav. Neurosci.* 19, 1-39. 10.3758/s13415-018-00653-4 30361863

[DMM052324C20] De Giorgio, F., Maduro, C., Fisher, E. M. C. and Acevedo-Arozena, A. (2019). Transgenic and physiological mouse models give insights into different aspects of amyotrophic lateral sclerosis. *Dis. Model. Mech.* 12, dmm037424. 10.1242/dmm.037424 30626575 PMC6361152

[DMM052324C21] De La Zerda, S. H., Netser, S., Magalnik, H., Briller, M., Marzan, D., Glatt, S., Abergel, Y. and Wagner, S. (2022). Social recognition in laboratory mice requires integration of behaviorally-induced somatosensory, auditory and olfactory cues. *Psychoneuroendocrinology* 143, 105859. 10.1016/j.psyneuen.2022.105859 35816892

[DMM052324C22] Deacon, R. M. (2006). Assessing nest building in mice. *Nat. Protoc.* 1, 1117-1119. 10.1038/nprot.2006.170 17406392

[DMM052324C23] DeJesus-Hernandez, M., MacKenzie, I. R., Boeve, B. F., Boxer, A. L., Baker, M., Rutherford, N. J., Nicholson, A. M., Finch, N. A., Flynn, H., Adamson, J. et al. (2011). Expanded GGGGCC hexanucleotide repeat in noncoding region of C9ORF72 causes chromosome 9p-Linked FTD and ALS. *Neuron* 72, 245-256. 10.1016/j.neuron.2011.09.011 21944778 PMC3202986

[DMM052324C24] Douglas, R. M., Alam, N. M., Silver, B. D., McGill, T. J., Tschetter, W. W. and Prusky, G. T. (2005). Independent visual threshold measurements in the two eyes of freely moving rats and mice using a virtual-reality optokinetic system. *Vis. Neurosci.* 22, 677-684. 10.1017/S0952523805225166 16332278

[DMM052324C25] Fisher, E. M. C., Greensmith, L., Malaspina, A., Fratta, P., Hanna, M. G., Schiavo, G., Isaacs, A. M., Orrell, R. W., Cunningham, T. J. and Arozena, A. A. (2023). Opinion: more mouse models and more translation needed for ALS. *Mol. Neurodegener.* 18, 30. 10.1186/s13024-023-00619-2 37143081 PMC10161557

[DMM052324C26] Gregory, J. M., McDade, K., Bak, T. H., Pal, S., Chandran, S., Smith, C. and Abrahams, S. (2020). Executive, language and fluency dysfunction are markers of localised TDP-43 cerebral pathology in non-demented ALS. *J. Neurol. Neurosurg. Psychiatry* 91, 149-157. 10.1136/jnnp-2019-320807 31515300 PMC6996101

[DMM052324C27] Hao, Z., Liu, L., Tao, Z., Wang, R., Ren, H., Sun, H., Lin, Z., Zhang, Z., Mu, C., Zhou, J. et al. (2019). Motor dysfunction and neurodegeneration in a C9orf72 mouse line expressing poly-PR. *Nat. Commun.* 10, 2906. 10.1038/s41467-019-10956-w 31266945 PMC6606620

[DMM052324C28] Hardiman, O., Al-Chalabi, A., Chio, A., Corr, E. M., Logroscino, G., Robberecht, W., Shaw, P. J., Simmons, Z. and Van Den Berg, L. H. (2017). Amyotrophic lateral sclerosis. *Nat. Rev. Dis. Primers* 3, 17071. 10.1038/nrdp.2017.71 28980624

[DMM052324C29] Harms, M. B., Cady, J., Zaidman, C., Cooper, P., Bali, T., Allred, P., Cruchaga, C., Baughn, M., Libby, R. T., Pestronk, A. et al. (2013). Lack of C9ORF72 coding mutations supports a gain of function for repeat expansions in amyotrophic lateral sclerosis. *Neurobiol. Aging* 34, 2234.e13-2234.e19. 10.1016/j.neurobiolaging.2013.03.006 PMC3679344 23597494

[DMM052324C30] Irwin, D. J., McMillan, C. T., Brettschneider, J., Libon, D. J., Powers, J., Rascovsky, K., Toledo, J. B., Boller, A., Bekisz, J., Chandrasekaran, K. et al. (2013). Cognitive decline and reduced survival in *C9orf72* expansion frontotemporal degeneration and amyotrophic lateral sclerosis. *J. Neurol. Neurosurg. Psychiatry* 84, 163-169. 10.1136/jnnp-2012-303507 23117491 PMC3543474

[DMM052324C31] Jhuang, H., Garrote, E., Yu, X., Khilnani, V., Poggio, T., Steele, A. D. and Serre, T. (2010). Automated home-cage behavioural phenotyping of mice. *Nat. Commun.* 1, 68. 10.1038/ncomms1064 20842193

[DMM052324C32] Jiang, J., Zhu, Q., Gendron, T. F., Saberi, S., McAlonis-Downes, M., Seelman, A., Stauffer, J. E., Jafar-nejad, P., Drenner, K., Schulte, D. et al. (2016). Gain of toxicity from ALS/FTD-linked repeat expansions in C9ORF72 is alleviated by antisense oligonucleotides targeting GGGGCC-containing RNAs. *Neuron* 90, 535-550. 10.1016/j.neuron.2016.04.006 27112497 PMC4860075

[DMM052324C33] Kabashi, E., Valdmanis, P. N., Dion, P., Spiegelman, D., McConkey, B. J., Vande Velde, C., Bouchard, J.-P., Lacomblez, L., Pochigaeva, K., Salachas, F. et al. (2008). TARDBP mutations in individuals with sporadic and familial amyotrophic lateral sclerosis. *Nat. Genet.* 40, 572-574. 10.1038/ng.132 18372902

[DMM052324C34] Keszycki, R., Rodriguez, G., Dunn, J. T., Locci, A., Orellana, H., Haupfear, I., Dominguez, S., Fisher, D. W. and Dong, H. (2023). Characterization of apathy-like behaviors in the 5xFAD mouse model of Alzheimer's disease. *Neurobiol. Aging* 126, 113-122. 10.1016/j.neurobiolaging.2023.02.012 36989547 PMC10106415

[DMM052324C35] Kim, E., White, M. A., Phillips, B. U., Lopez-Cruz, L., Kim, H., Heath, C. J., Lee, J. E., Saksida, L. M., Sreedharan, J. and Bussey, T. J. (2020). Coexistence of perseveration and apathy in the TDP-43Q331K knock-in mouse model of ALS–FTD. *Transl. Psychiatry* 10, 377. 10.1038/s41398-020-01078-9 33149110 PMC7643138

[DMM052324C36] Kuznetsova, A., Brockhoff, P. B. and Christensen, R. H. B. (2017). lmerTest package: tests in linear mixed effects models. *J. Stat. Soft.* 82, 1-26. 10.18637/jss.v082.i13

[DMM052324C37] LaClair, K. D., Zhou, Q., Michaelsen, M., Wefers, B., Brill, M. S., Janjic, A., Rathkolb, B., Farny, D., Cygan, M., De Angelis, M. H. et al. (2020). Congenic expression of poly-GA but not poly-PR in mice triggers selective neuron loss and interferon responses found in C9orf72 ALS. *Acta Neuropathol.* 140, 121-142. 10.1007/s00401-020-02176-0 32562018 PMC7360660

[DMM052324C38] Lipina, T., Men, X., Blundell, M., Salahpour, A. and Ramsey, A. J. (2022). Abnormal sensory perception masks behavioral performance of Grin1 knockdown mice. *Genes Brain Behav.* 21, e12825. 10.1111/gbb.12825 35705513 PMC9744498

[DMM052324C39] Liu, Y., Pattamatta, A., Zu, T., Reid, T., Bardhi, O., Borchelt, D. R., Yachnis, A. T. and Ranum, L. P. W. (2016). C9orf72 BAC mouse model with motor deficits and neurodegenerative features of ALS/FTD. *Neuron* 90, 521-534. 10.1016/j.neuron.2016.04.005 27112499

[DMM052324C40] Meneses, A., Koga, S., O'Leary, J., Dickson, D. W., Bu, G. and Zhao, N. (2021). TDP-43 pathology in Alzheimer's disease. *Mol. Neurodegener.* 16, 84. 10.1186/s13024-021-00503-x 34930382 PMC8691026

[DMM052324C41] Milioto, C., Carcolé, M., Giblin, A., Coneys, R., Attrebi, O., Ahmed, M., Harris, S. S., Lee, B. I., Yang, M., Ellingford, R. A. et al. (2024). PolyGR and polyPR knock-in mice reveal a conserved neuroprotective extracellular matrix signature in C9orf72 ALS/FTD neurons. *Nat. Neurosci.* 27, 643-655. 10.1038/s41593-024-01589-4 38424324 PMC11001582

[DMM052324C42] Mizielinska, S., Grönke, S., Niccoli, T., Ridler, C. E., Clayton, E. L., Devoy, A., Moens, T., Norona, F. E., Woollacott, I. O. C., Pietrzyk, J. et al. (2014). *C9orf72* repeat expansions cause neurodegeneration in *Drosophila* through arginine-rich proteins. *Science* 345, 1192-1194. 10.1126/science.1256800 25103406 PMC4944841

[DMM052324C43] Moens, T. G., Da Cruz, S., Neumann, M., Shelkovnikova, T. A., Shneider, N. A. and Van Den Bosch, L. (2025). Amyotrophic lateral sclerosis caused by FUS mutations: advances with broad implications. *Lancet Neurol.* 24, 166-178. 10.1016/S1474-4422(24)00517-9 39862884

[DMM052324C44] Moglia, C., Calvo, A., Canosa, A., Manera, U., Vasta, R., Di Pede, F., Daviddi, M., Matteoni, E., Brunetti, M., Sbaiz, L. et al. (2024). Cognitive and behavioral features of patients with amyotrophic lateral sclerosis who are carriers of the *TARDBP* pathogenic variant. *Neurology* 102, e208082. 10.1212/WNL.0000000000208082 38261982 PMC10962913

[DMM052324C45] Neumann, M., Sampathu, D. M., Kwong, L. K., Truax, A. C., Micsenyi, M. C., Chou, T. T., Bruce, J., Schuck, T., Grossman, M., Clark, C. M. et al. (2006). Ubiquitinated TDP-43 in frontotemporal lobar degeneration and amyotrophic lateral sclerosis. *Science* 314, 130-133. 10.1126/science.1134108 17023659

[DMM052324C46] Olney, N. T., Spina, S. and Miller, B. L. (2017). Frontotemporal dementia. *Neurol. Clin.* 35, 339-374. 10.1016/j.ncl.2017.01.008 28410663 PMC5472209

[DMM052324C47] O'Rourke, J. G., Bogdanik, L., Muhammad, A. K. M. G., Gendron, T. F., Kim, K. J., Austin, A., Cady, J., Liu, E. Y., Zarrow, J., Grant, S. et al. (2015). C9orf72 BAC transgenic mice display typical pathologic features of ALS/FTD. *Neuron* 88, 892-901. 10.1016/j.neuron.2015.10.027 26637796 PMC4672384

[DMM052324C48] Oummadi, A., Meyer-Dilhet, G., Béry, A., Aubert, A., Barone, P., Mortaud, S., Guillemin, G. J., Menuet, A. and Laugeray, A. (2020). 3Rs-based optimization of mice behavioral testing: the habituation/dishabituation olfactory test. *J. Neurosci. Methods* 332, 108550. 10.1016/j.jneumeth.2019.108550 31838181

[DMM052324C49] Peters, O. M., Cabrera, G. T., Tran, H., Gendron, T. F., McKeon, J. E., Metterville, J., Weiss, A., Wightman, N., Salameh, J., Kim, J. et al. (2015). Human C9ORF72 hexanucleotide expansion reproduces RNA foci and dipeptide repeat proteins but not neurodegeneration in BAC transgenic mice. *Neuron* 88, 902-909. 10.1016/j.neuron.2015.11.018 26637797 PMC4828340

[DMM052324C50] Poos, J. M., MacDougall, A., Van Den Berg, E., Jiskoot, L. C., Papma, J. M., Van Der Ende, E. L., Seelaar, H., Russell, L. L., Peakman, G., Convery, R. et al. (2022). Longitudinal cognitive changes in genetic frontotemporal dementia within the GENFI cohort. *Neurology* 99, e281-e295. 10.1212/WNL.0000000000200384 35483895 PMC9302936

[DMM052324C51] Renton, A. E., Majounie, E., Waite, A., Simón-Sánchez, J., Rollinson, S., Gibbs, J. R., Schymick, J. C., Laaksovirta, H., van Swieten, J. C., Myllykangas, L. et al. (2011). A hexanucleotide repeat expansion in C9ORF72 is the cause of chromosome 9p21-linked ALS-FTD. *Neuron* 72, 257-268. 10.1016/j.neuron.2011.09.010 21944779 PMC3200438

[DMM052324C52] Reynolds, T. H., Dalton, A., Calzini, L., Tuluca, A., Hoyte, D. and Ives, S. J. (2019). The impact of age and sex on body composition and glucose sensitivity in C57BL/6J mice. *Physiol. Rep.* 7, e13995. 10.14814/phy2.13995 30706674 PMC6356156

[DMM052324C53] Rusina, R., Vandenberghe, R. and Bruffaerts, R. (2021). Cognitive and behavioral manifestations in ALS: beyond motor system involvement. *Diagnostics* 11, 624. 10.3390/diagnostics11040624 33808458 PMC8065866

[DMM052324C54] Sanderson, D. J., Good, M. A., Skelton, K., Sprengel, R., Seeburg, P. H., Rawlins, J. N. P. and Bannerman, D. M. (2009). Enhanced long-term and impaired short-term spatial memory in GluA1 AMPA receptor subunit knockout mice: evidence for a dual-process memory model. *Learn. Mem.* 16, 379-386. 10.1101/lm.1339109 19470654 PMC2704103

[DMM052324C55] Schönecker, S., Martinez-Murcia, F. J., Denecke, J., Franzmeier, N., Danek, A., Wagemann, O., Prix, C., Wlasich, E., Vöglein, J., Loosli, S. V. et al. (2024). Frequency and longitudinal course of behavioral and neuropsychiatric symptoms in participants with genetic frontotemporal dementia. *Neurology* 103, e209569. 10.1212/WNL.0000000000209569 39284109 PMC11399068

[DMM052324C56] Sreedharan, J., Blair, I. P., Tripathi, V. B., Hu, X., Vance, C., Rogelj, B., Ackerley, S., Durnall, J. C., Williams, K. L., Buratti, E. et al. (2008). TDP-43 mutations in familial and sporadic amyotrophic lateral sclerosis. *Science* 319, 1668-1672. 10.1126/science.1154584 18309045 PMC7116650

[DMM052324C57] Stallings, N. R., Puttaparthi, K., Dowling, K. J., Luther, C. M., Burns, D. K., Davis, K. and Elliott, J. L. (2013). TDP-43, an ALS linked protein, regulates fat deposition and glucose homeostasis. *PLoS ONE* 8, e71793. 10.1371/journal.pone.0071793 23967244 PMC3742534

[DMM052324C58] Strong, M. J., Abrahams, S., Goldstein, L. H., Woolley, S., McLaughlin, P., Snowden, J., Mioshi, E., Roberts-South, A., Benatar, M., HortobáGyi, T. et al. (2017). Amyotrophic lateral sclerosis - frontotemporal spectrum disorder (ALS-FTSD): revised diagnostic criteria. *Amyotroph. Lateral Scler. Frontotemporal Degener.* 18, 153-174. 10.1080/21678421.2016.1267768 28054827 PMC7409990

[DMM052324C59] Sudria-Lopez, E., Koppers, M., De Wit, M., Van Der Meer, C., Westeneng, H.-J., Zundel, C. A. C., Youssef, S. A., Harkema, L., De Bruin, A., Veldink, J. H. et al. (2016). Full ablation of C9orf72 in mice causes immune system-related pathology and neoplastic events but no motor neuron defects. *Acta Neuropathol.* 132, 145-147. 10.1007/s00401-016-1581-x 27206760 PMC4911370

[DMM052324C60] Sukoff Rizzo, S. J., Anderson, L. C., Green, T. L., McGarr, T., Wells, G. and Winter, S. S. (2018). Assessing healthspan and lifespan measures in aging mice: optimization of testing protocols, replicability, and rater reliability. *Curr. Protoc. Mouse Biol.* 8, e45. 10.1002/cpmo.45 29924918

[DMM052324C61] Todd, T. W. and Petrucelli, L. (2022). Modelling amyotrophic lateral sclerosis in rodents. *Nat. Rev. Neurosci.* 23, 231-251. 10.1038/s41583-022-00564-x 35260846

[DMM052324C62] Verdone, B. M., Cicardi, M. E., Wen, X., Sriramoji, S., Russell, K., Markandaiah, S. S., Jensen, B. K., Krishnamurthy, K., Haeusler, A. R., Pasinelli, P. et al. (2022). A mouse model with widespread expression of the C9orf72-linked glycine–arginine dipeptide displays non-lethal ALS/FTD-like phenotypes. *Sci. Rep.* 12, 5644. 10.1038/s41598-022-09593-z 35379876 PMC8979946

[DMM052324C63] Watkins, J. A., Alix, J. J. P., Shaw, P. J. and Mead, R. J. (2021). Extensive phenotypic characterisation of a human TDP-43Q331K transgenic mouse model of amyotrophic lateral sclerosis (ALS). *Sci. Rep.* 11, 16659. 10.1038/s41598-021-96122-z 34404845 PMC8370970

[DMM052324C64] Wen, X., Tan, W., Westergard, T., Krishnamurthy, K., Markandaiah, S. S., Shi, Y., Lin, S., Shneider, N. A., Monaghan, J., Pandey, U. B. et al. (2014). Antisense proline-arginine RAN dipeptides linked to C9ORF72-ALS/FTD form toxic nuclear aggregates that initiate in vitro and in vivo neuronal death. *Neuron* 84, 1213-1225. 10.1016/j.neuron.2014.12.010 25521377 PMC4632245

[DMM052324C65] White, M. A., Kim, E., Duffy, A., Adalbert, R., Phillips, B. U., Peters, O. M., Stephenson, J., Yang, S., Massenzio, F., Lin, Z. et al. (2018). TDP-43 gains function due to perturbed autoregulation in a Tardbp knock-in mouse model of ALS-FTD. *Nat. Neurosci.* 21, 552-563. 10.1038/s41593-018-0113-5 29556029 PMC5884423

[DMM052324C66] Whitehead, J. C., Hildebrand, B. A., Sun, M., Rockwood, M. R., Rose, R. A., Rockwood, K. and Howlett, S. E. (2014). A clinical frailty index in aging mice: comparisons with frailty index data in humans. *J. Gerontol. Ser. A* 69, 621-632. 10.1093/gerona/glt136 PMC4022099 24051346

[DMM052324C67] Zhang, Y.-J., Gendron, T. F., Ebbert, M. T. W., O'Raw, A. D., Yue, M., Jansen-West, K., Zhang, X., Prudencio, M., Chew, J., Cook, C. N. et al. (2018). Poly(GR) impairs protein translation and stress granule dynamics in C9orf72-associated frontotemporal dementia and amyotrophic lateral sclerosis. *Nat. Med.* 24, 1136-1142. 10.1038/s41591-018-0071-1 29942091 PMC6520050

[DMM052324C68] Zhang, Y.-J., Guo, L., Gonzales, P. K., Gendron, T. F., Wu, Y., Jansen-West, K., O'Raw, A. D., Pickles, S. R., Prudencio, M., Carlomagno, Y. et al. (2019). Heterochromatin anomalies and double-stranded RNA accumulation underlie C9orf72 poly(PR) toxicity. *Science* 363, eaav2606. 10.1126/science.aav2606 30765536 PMC6524780

