# Supplementary material for: Multi-modal comparative phenotyping of knock-in mouse models of frontotemporal dementia/amyotrophic lateral sclerosis
Source: Dis Model Mech. 2025 Aug 26;18(8):dmm052324. doi: 10.1242/dmm.052324 (PMC12421800; doi:10.1242/dmm.052324)
Supplement: Supplementary information [file dmm-18-052324-s1.pdf]

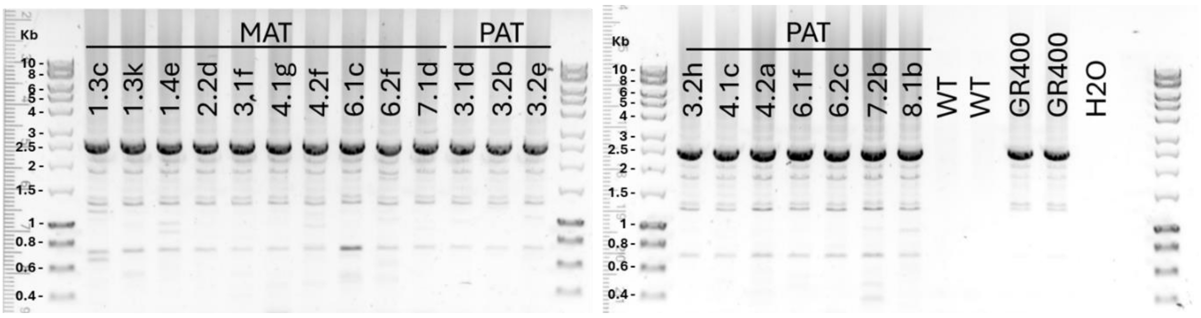

Fig. S1. **PCR analysis of repeat expansion length in the brain of *C9orf72*$^{GR400/+}$ mice, showing a ~2.5 Kb PCR product in *C9orf72*$^{GR400/+}$ animals that inherited their mutant allele from their mothers (MAT) and fathers (PAT), which is the same size as that amplified from reference DNA (GR400).** No such product is produced in the absence of DNA (H20) or from wildtype (WT) controls.

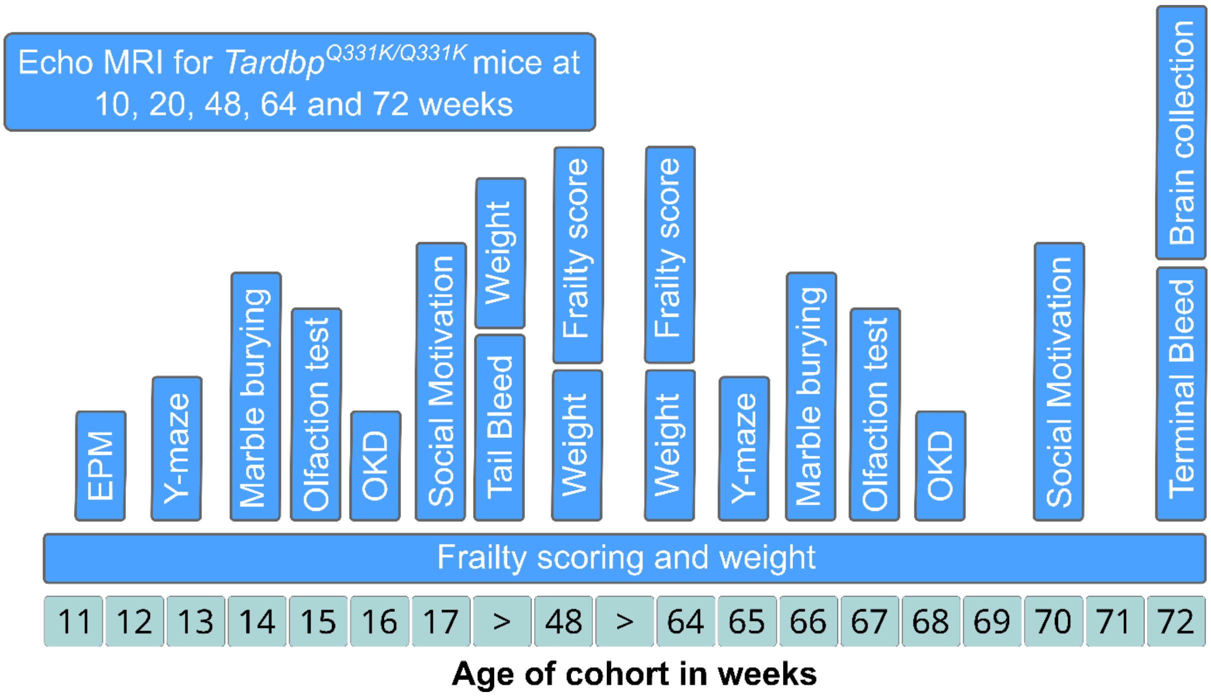

Fig. S2. **Timeline of behavioural tests performed in the *C9orf72*^{GR400} and the *Tardbp*^{Q331K} study. elevated plus maze (EPM), optokinetic drum (OKD), magnetic resonance imaging (MRI).**

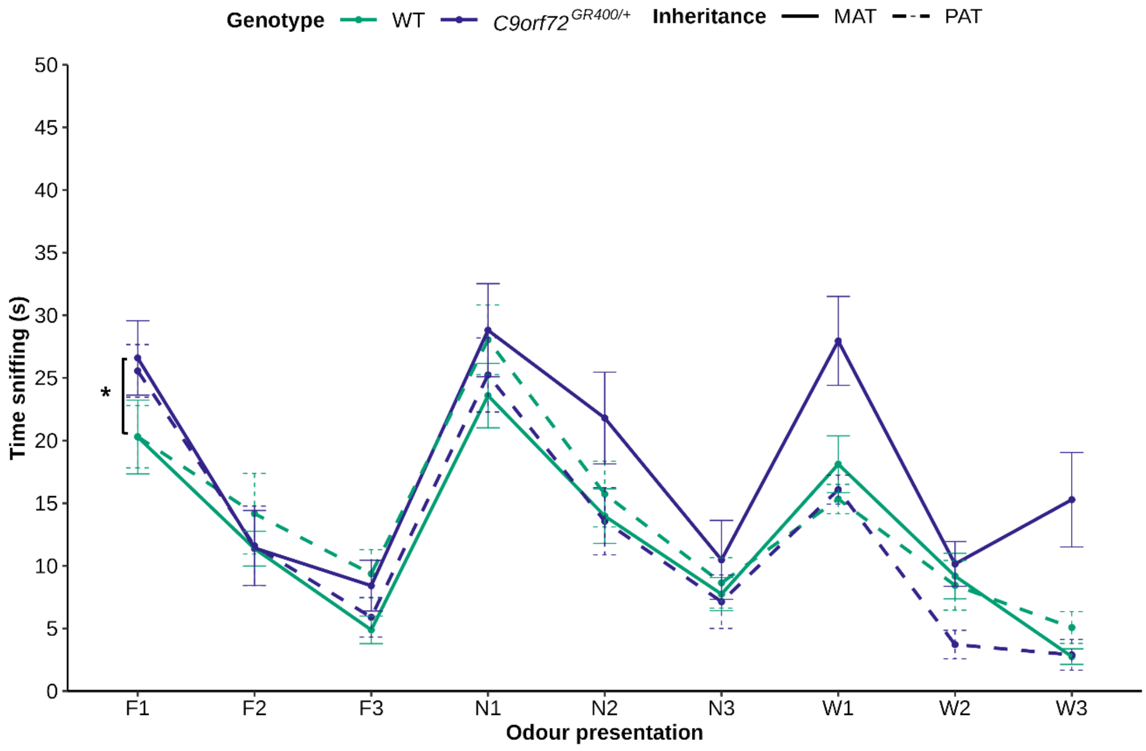

Fig. S3. Assessment of sensory function in the *C9orf72*$^{GR400/+}$ mouse model. Sniffing time for the first, second and third presentations of familiar social odour (F1, F2, F3), novel social odour (N1, N2, N3) and water odour (W1, W2, W3), solid line – maternal inheritance of mutant allele, dashed line – paternal inheritance. Significant difference in the sniffing time between WT and *C9orf72*$^{GR400/+}$ in the maternal inheritance group at F1 ($p=0.0322$), $p<0.05$=*. Error bars represent mean ± SEM. For olfaction, each point shows average time sniffing ± SEM for each genotype group, each mouse was tested once at every odour presentation. Videos scored: WT n=24, *C9orf72*$^{GR400/+}$ n=24.

Disease Models & Mechanisms • Supplementary information

Table S1. Number of mice used for the analysis in each test at each time point. Mice were excluded from analysis *only* if they had to be culled for welfare reasons unless otherwise specified.

WT vs *C9orf72*$^{GR400/+}$ study

**Behavioural assay**	**Young time point (average)**	**Number of mice**	**Old time point (average)**	**Number of mice**
Elevated plus maze	11-12 weeks	WT=24 (female MAT=6, female PAT=6, male MAT=6, male PAT = 6) *C9orf72*$^{GR400/+}$=24 (female MAT=6, female PAT=6, male MAT=6, male PAT = 6)	NA	NA
Y-maze (total distance and novel preference ratio)	12.5 weeks	WT=21 (female MAT=5, female PAT=6, male MAT=4, male PAT = 6) *C9orf72*$^{GR400/+}$=20 (female MAT=5, female PAT=5, male MAT=5, male PAT = 5) *7 mice excluded due to jumping out of the arena (C9ORF72-GR400-MAT-B6J/7.1g, C9ORF72-GR400-MAT-B6J/6.2c, C9ORF72-GR400-MAT-B6J/7.1d, C9ORF72-GR400-PAT-B6J/8.1b, C9ORF72-GR400-MAT-B6J/6.1c, C9ORF72-GR400-PAT-B6J/4.1c,C9ORF72-GR400-MAT-B6J/4.2g)*	64.5 weeks	WT=21(female MAT=6, female PAT=6, male MAT=6, male PAT = 3) *C9orf72*$^{GR400/+}$=20 (female MAT=5, female PAT=6, male MAT=6, male PAT = 3)
Marble burying test	14 weeks	WT=24 (female MAT=6, female PAT=6, male MAT=6, male PAT = 6) *C9orf72*$^{GR400/+}$=24 (female MAT=6, female PAT=6, male MAT=6, male PAT = 6)	66.5 weeks	WT=20 (female MAT = 6, female PAT = 5, male MAT = 6, male PAT = 3) *C9orf72*$^{GR400/+}$=20 (female MAT = 5, female PAT = 6, male MAT = 6, male PAT = 3)
Olfaction test (videos scored)	15 weeks	WT=24 (female MAT=6, female PAT=6, male MAT=6, male PAT = 6) *C9orf72*$^{GR400/+}$=24 (female MAT=6, female PAT=6,	67 weeks	WT=20 (female MAT=6, female PAT=5, male MAT=6, male PAT = 3) *C9orf72*$^{GR400/+}$=18 (female MAT=3, female PAT=6,

Disease Models & Mechanisms • Supplementary information

		male MAT=6, male PAT = 6)		male MAT=6, male PAT = 3)<br>*2 videos excluded due to procedural failure; wrong hopper used (C9ORF72-GR400-MAT-B6J/1.3c, C9ORF72-GR400-MAT-B6J/2.2d)*
Optokinetic drum	15 weeks	WT=23 (female MAT=6, female PAT=6, male MAT=6, male PAT = 5)<br>*C9orf72*$^{GR400/+}$=23 (female MAT=6, female PAT=6, male MAT=6, male PAT = 5)<br>*2 mice excluded, 1 due to corrupted data file (C9ORF72-GR400-PAT-B6J/3.2d), 1 due to being uncooperative during test; jumping off the OKD platform (C9ORF72-GR400-PAT-B6J/7.2h).*	68 weeks	WT=20 (female MAT=6, female PAT=5, male MAT=6, male PAT = 3)<br>*C9orf72*$^{GR400/+}$=20 (female MAT=5, female PAT=6, male MAT=6, male PAT = 3)
Social motivation test	18.5 weeks	WT=22 (female MAT=6, female PAT=6, male MAT=5, male PAT = 5)<br>*C9orf72*$^{GR400/+}$=24 (female MAT=6, female PAT=6, male MAT=6, male PAT = 6)<br>*2 mice excluded – uncooperative during the test C9ORF72-GR400-MAT-B6J/4.2g, C9ORF72-GR400-PAT-B6J/7.2e – sitting on top of object*	70 weeks	WT=20 (female MAT=6, female PAT=5, male MAT=6, male PAT = 3)<br>*C9orf72*$^{GR400/+}$=19 (female MAT=5, female PAT=6, male MAT=5, male PAT = 3)

WT vs *Tardbp*$^{Q331K/Q331K}$ study

**Behavioural assay**	**Young time point (average)**	**Number of mice**	**Old time point (average)**	**Number of mice**
Elevated plus maze	11-12 weeks	WT = 27 (female = 12, males = 15)<br>*Tardbp*$^{Q331K/Q331K}$ = 26 (female = 11, male = 15)<br>*1 mouse excluded from the analysis due to poor video tracking during test such that only 21.04 s out of 300 s of performance could be monitored (TDP-43-Q331K-B6J-IC/1.1c)*	NA	NA

Disease Models & Mechanisms • Supplementary information

Y-maze (total distance)	12.5 weeks	WT = 27 (female = 12, males = 15) *Tardbp*$^{Q331K/Q331K}$ = 27 (female = 12, male = 15)	65.5 weeks	WT=20 (female=11, male=9), *Tardbp*$^{Q331K/Q331K}$ = 21 (female=11, male=10) *1 mouse removed, did not leave start arm (TDP-43-Q331K-B6J-IC/3.3n)*
Y-maze (novel preference ratio)	12.5 weeks	WT = 27 (female = 12, males = 15) *Tardbp*$^{Q331K/Q331K}$ = 26 (female = 11, male = 15) *1 mouse removed, did not leave start arm during test phase (TDP-43-Q331K-B6J-IC/5.1f – not included in NPR analysis - NA)*	65.5 weeks	WT=19 (female=10, male=9), *Tardbp*$^{Q331K/Q331K}$ = 21 (female=11, male=10) *2 mice removed, 1 did not leave start arm (TDP-43-Q331K-B6J-IC/3.3n – removed from all testing), 1 did not leave start arm during test phase (TDP-43-Q331K-B6J-IC/3.2a – not included in NPR analysis)*
Marble burying test	14 weeks	WT = 27 (female = 12, males = 15) *Tardbp*$^{Q331K/Q331K}$ = 27 (female = 12, male = 15)	67 weeks	WT = 21 (female=11, male=10) *Tardbp*$^{Q331K/Q331K}$ = 20 (female=11, male=9)
Olfaction test (videos scored)	15 weeks	WT = 27 (female = 12, males = 15), *Tardbp*$^{Q331K/Q331K}$ = 24 (female = 9, male = 15) *3 mice excluded due to wrong order of smells presentation during the test, treated as procedural failure (TDP43-Q331K-B6J-IC/1.1c, TDP43-Q331K-B6J-IC/4.1a, TDP43-Q331K-B6J-IC/6.1d).*	67 weeks	WT = 21 (female = 11, males = 10), *Tardbp*$^{Q331K/Q331K}$ = 20 (female = 11, male = 9) *1 mouse excluded due to corrupted video file (TDP43-Q331K-B6J-IC/4.2e)*
Optokinetic drum	16 weeks	WT = 27 (female = 12, males = 15) *Tardbp*$^{Q331K/Q331K}$ = 26 (female = 11, male = 15) *1 mouse excluded due to procedural failure, video file not acquired (TDP-43-Q331K-B6J-IC/1.1b).*	68 weeks	WT = 21 (female = 11, males = 10), *Tardbp*$^{Q331K/Q331K}$ = 20 (female = 11, male = 9)
Social motivation test	18 weeks	WT = 27 (female = 12, males = 15) *Tardbp*$^{Q331K/Q331K}$ = 27 (female = 12, male = 15)	71 weeks	WT = 20 (female = 10, males = 10), *Tardbp*$^{Q331K/Q331K}$ = 20 (female = 11, male = 9)

**Echo-MRI/ age (weeks)**	**WT**	***Tardbp*$^{Q331K/Q331K}$**

Disease Models & Mechanisms • Supplementary information

10	27 (female = 12, males = 15)	27 (female = 12, male = 15)
20	27 (female = 12, males = 15)	27 (female = 12, male = 15)
48	23 (female = 12, males = 11)	26 (female = 12, male = 14)
64	21 (female = 11, males = 10)	22 (female = 12, male = 10)
72	20 (female = 10, males = 10)	20 (female = 11, male = 9)

Table S2. Number of animals used for the weight study for each age – 4 to 72 weeks.

A: *C9orf72* study

Age in weeks	WT female (n)	WT Male (n)	*C9orf72*$^{GR400/+}$ Female (n)	*C9orf72*$^{GR400/+}$ Male (n)
4	12	12	12	12
5	6	0	4	2
6	4	6	5	6
8	12	12	12	12
9	10	12	11	12
10	12	12	12	12
12	6	9	8	9
13	8	3	5	3
14	7	11	9	9
16	12	12	12	12
17	6	9	8	9
18	7	9	7	9
20	12	12	12	12
21	7	11	9	9
22	11	10	11	12
23	12	7	10	9
24	12	9	10	9
26	7	11	9	9
27	7	6	8	7
28	12	12	12	12
30	8	11	10	11
31	11	12	10	11
32	11	12	12	11
34	7	6	7	7
35	10	7	8	7
36	12	12	12	12
38	2	5	4	5
39	11	12	10	10
42	11	9	10	10
43	7	6	7	7
44	5	3	5	5
46	12	9	12	12
47	9	6	7	5
48	8	9	10	12

Disease Models & Mechanisms • Supplementary information

50	10	7	8	7
51	7	7	9	8
52	8	9	11	12
54	7	8	9	9
55	10	6	8	6
56	9	9	10	11
57	6	1	4	2
58	10	8	10	10
60	12	6	9	8
61	10	6	8	6
62	9	8	9	9
64	8	9	9	9
65	8	1	4	3
66	11	9	11	9
68	10	9	10	9
69	6	1	6	4
70	11	9	9	7

B: *Tardp* study

Age in weeks	WT female (n)	WT Male (n)	*Tardbp*$^{Q331K/Q331K}$ Female (n)	*Tardbp*$^{Q331K/Q331K}$ Male (n)
4	12	15	12	15
5	12	15	12	15
6	12	15	12	15
8	8	9	8	12
10	12	15	12	15
11	12	14	11	15
12	10	15	11	15
14	12	12	12	12
15	8	3	7	9
16	12	15	12	15
19	8	12	9	12
20	12	15	12	15
22	8	6	7	12
24	11	15	11	15
25	11	15	12	15
28	12	15	12	14
29	6	9	6	3
30	12	14	12	14
32	12	13	11	14
33	6	9	6	3
34	12	11	11	14
36	12	12	12	14
38	12	12	12	14
39	4	8	4	3
40	12	12	12	14
43	12	12	12	14
44	6	9	6	3
48	12	12	12	14
50	4	8	5	3
52	12	10	12	14

54	12	10	12	14
56	10	10	12	14
58	12	10	12	13
60	12	10	12	11
62	12	10	12	10
63	12	10	12	10
64	11	9	11	10
66	11	10	11	10
67	11	10	11	9
68	11	10	11	9
70	11	10	11	9
72	11	10	11	9

Table S3. Olfactory habituation-dishabituation statistics – blue cells show normal habituation, purple cells show normal dishabituation to familiar and novel mouse odours.

A:

**WT vs *C9orf72$^{GR400/+}$* 15 weeks,** significant main effect of odour (smell) F(8, 271.407)=36.2374),<br>p<2e-16<br>significant interaction between genotype and inheritance F(1, 39.181=5.2138), p=0.02791		
Bonferroni correction: pairwise ~ smell|genotype		
Mouse group	Smell and presentation comparison	P value
WT	familiar smell presentation 1 (F1) vs familiar smell presentation 3 (F3)	P<0.0001
	novel smell presentation 1 (N1) vs novel smell presentation 3 (N3)	P<0.0001
	water presentation 1 (W1) vs water presentation 3 (W3)	P=0.0002
	W3 – F1	P<0.0001
	W3 – N1	P<0.0001
	F3 – N1	P<0.0001
	F3 – W1	P=0.0364
	N3 – F1	P=0.0003
	N3 – W1	P=0.1686 (ns)
C9orf72$^{GR400/+}$	F1 – F3	P<0.0001
	N1 – N3	P<0.0001
	W1 – W3	P=0.0017
	W3 – F1	P<0.0001
	W3 – N1	P<0.0001
	F3 – N1	P<0.0001
	F3 – W1	P=0.0001
	N3 – F1	P<0.0001
	N3 – W1	P=0.0008

B:

**WT vs *C9orf72*$^{GR400/+}$ 67 weeks,** significant main effect of odour (smell) (F(8,251.857)=18.7708), p<2e-16		
Bonferroni correction: pairwise ~ smell|genotype		
Mouse group	Smell and presentation comparison	P value
WT	F1 – F3	P<0.0001
	N1 – N3	P=0.0351
	W1 – W3	ns (P=0.9468)
	W3 – F1	P=0.0017
	W3 – N1	P=0.0001
	F3 – N1	P<0.0001
	F3 – W1	ns (P=0.1783)
	N3 – F1	ns (0.3449)
	N3 – W1	ns (1)
C9orf72$^{GR400/+}$	F1 – F3	P<0.0001
	N1 – N3	P<0.0001
	W1 – W3	P=0.0010
	W3 – F1	P<0.0001
	W3 – N1	P<0.0001
	F3 – N1	P<0.0001
	F3 – W1	P=0.0323
	N3 – F1	P=0.0142
	N3 – W1	ns (p=1)

C:

**WT vs *Tardbp*$^{Q331K/Q331K}$ 15 weeks,** significant main effect of odour (smell) (F(8,282.464)=30.8712), p<2e-16		
Bonferroni correction: pairwise ~ smell|genotype		
Mouse group	Smell and presentation comparison	P value
WT	F1 – F3	P<0.0001
	N1 – N3	P=0.0003
	W1 – W3	P<0.0001
	W3 – F1	P<0.0001
	W3 – N1	P<0.0001
	F3 – N1	P<0.0001
	F3 – W1	ns P=0.0708
	N3 – F1	P<0.0001
	N3 – W1	ns P=0.8087
Tardbp$^{Q331K/Q331K}$	F1 – F3	P<0.0001
	N1 – N3	P<0.0001
	W1 – W3	P=0.0022
	W3 – F1	P<0.0001
	W3 – N1	P<0.0001
	F3 – N1	P<0.0001
	F3 – W1	P=0.0318
	N3 – F1	P<0.0001
	N3 – W1	P=0.0079

Disease Models & Mechanisms • Supplementary information

D:

**WT vs *Tardbp*$^{Q331K/Q331K}$ 67 weeks,** significant main effect of odour (smell) (F(8,231.165)=27.3237), p<2e-16)		
Bonferroni correction: pairwise ~ smell|genotype		
Mouse group	Smell and presentation comparison	P value
WT	F1 – F3	P<0.0001
	N1 – N3	P<0.0001
	W1 – W3	ns P=0.5448
	W3 – F1	P<0.0001
	W3 – N1	P<0.0001
	F3 – N1	P<0.0001
	F3 – W1	ns (p=0.7869)
	N3 – F1	P<0.0001
	N3 – W1	ns (P=0.2135)
Tardbp$^{Q331K/Q331K}$	F1 – F3	P<0.0001
	N1 – N3	P=0.0035
	W1 – W3	P=0.0073
	W3 – F1	P<0.0001
	W3 – N1	P<0.0001
	F3 – N1	P<0.0001
	F3 – W1	ns (P=1)
	N3 – F1	P<0.0001
	N3 – W1	ns (p=1)

Table S4. Summary of results statistics *C9orf72*$^{GR400/+}$ study

Behavioural test	Statistical model<br>inh = inheritance	Significant main effects (F statistics)	Significant post hoc effects
**Weight**	averageweight~genotype*(age_weeks+sex+inh) + age_weeks*sex + (1|animal_name)	**age_weeks** F(50, 1571.27)= 265.4233, p < 2.2e-16 ***<br><br>**sex** F(1, 42.23)=53.1278, p=5.457e-09 ***<br><br>**age_weeks:sex** F(50, 1571.27)= 3.0632, p=1.107e-11 ***	**Compare sex (m vs f) at every age and genotype**<br><br>**F vs M in C9ORF72-GR400-B6J:WT** from 8 weeks: p=0.0154, stays significant for all ages onwards apart from 57, 68 and 69 weeks<br><br>**F vs M in C9ORF72-GR400-B6J:Het** from 8 weeks: p=0.0066, stays significant for all ages onwards apart from 68 weeks
**Olfaction test**	**Table 3 A and B**		
**Optokinetic drum (OKD)**	acuity~genotype*(sex + mean_experimental_age + inh) +(1|animal_name)	**mean_experimental_age** F (1, 40.863)=60.1028, p=1.49e-09 ***	**Compare age at every genotype**<br>**15 vs 68 weeks in C9ORF72-GR400-B6J:WT**<br>p<0.0001<br><br>**15 vs 68 weeks in C9ORF72-GR400-B6J:Het**<br>p<0.0001
**Elevated plus maze (EPM)**	**Duration**<br>Duration~genotype*(sex + inh + Section) + (1|animal_name)<br><br>**Frequency**<br>Frequency~genotype*(sex + inh + Section) + (1|animal_name)	**Duration**<br>**Section** F(2,134)=543.0345, p<2e-16 ***<br><br>**Frequency**<br>**Section** F(2, 92)=451.8957, p< 2.2e-16 ***<br><br>**genotype:Section** F(2, 92)= 7.9614, p=0.0006472 ***	**Duration**<br>No effects of interest to explore<br><br>**Frequency – compare genotype at every section**<br>**C9ORF72-GR400-B6J:WT vs C9ORF72-GR400-B6J:Het closed sections** p=0.1505<br><br>**C9ORF72-GR400-B6J:WT vs C9ORF72-GR400-B6J:Het open sections** p=0.5189<br><br>**C9ORF72-GR400-B6J:WT vs C9ORF72-GR400-B6J:Het centre** p=0.8911
**Marble burying**	kruskal.test<br>(marbles_burried ~ genotype)	**14 weeks**<br>Kruskal-Wallis chi-squared = 0.10584, df = 1, p-value = 0.7449<br><br>**66 weeks**<br>Kruskal-Wallis chi-squared = 0.80315, df = 1, p-value = 0.3702	NA
**Crawley three-chamber social preference test**	**Time spent with mouse**<br>time_mouse~genotype*(mean_experimental_age + sex + inh) + mean_experimental_age*inh + inh*sex + (1|animal_name)<br><br>**Time spent with object**<br>time_object~genotype*(mean_experimental_age + sex + inh) + inh*mean_experimental_age + (1|animal_name)<br><br>**Social preference ratio (SPR, based on time)**<br>SPRtime~genotype*(mean_experimental_age + sex + inh) + (1|animal_name)<br><br>**SPR (based on frequency)**<br>SPRfreq~genotype*(mean_experimental_age + sex + inh) + (1|animal_name)<br><br>**Distance travelled – habituation**<br>dist_hab_overall~genotype*(mean_experimental_age + sex + inh) + (1|animal_name)	**Time spent with mouse**<br>**mean_experimental_age** F(1, 39.274)=28.4675, p=4.244e-06 ***<br>**sex** (1, 38.207)= 5.9438, p=0.01953 *<br>**inh** F(1, 38.349)= 5.4988, p= 0.02430 *<br><br>**Time spent with object**<br>**mean_experimental_age** F(1, 36.704)= 6.9246, p=0.012357 *<br>**inh** F(1, 35.592)= 8.0067, p=0.007611 **<br><br>**SPR (based on time)**<br>**genotype:mean_experimental_age** F(1, 77)= 5.9038, p=0.01744 *<br><br>**SPR (based on frequency)**<br>**genotype:mean_experimental_age** F(1, 77)= 4.0001, p=0.04902 *<br><br>**Distance travelled - habituation**<br>**mean_experimental_age** F(1, 41.058)= 7.8548, p=0.007701 **	**Time spent with mouse – compare inheritance at every age**<br>**maternal vs paternal at 18.5 weeks**<br>p=0.0093<br>**maternal vs paternal at 70 weeks**<br>p=0.9085<br><br>**Time spent with mouse – compare sex at every inheritance**<br>**Male vs Female, maternal**<br>p=1<br>**Male vs Female, paternal**<br>p=0.0167<br><br>**Time spent with object – compare inheritance at every age**<br>**maternal vs paternal at 18.5 weeks**<br>p=0.0373<br>**maternal vs paternal at 70 weeks**<br>p=0.1390<br><br>**SPR (based on time) – compare genotype at every age**<br>**C9ORF72-GR400-B6J:WT vs C9ORF72-GR400-B6J:Het at 18.5 weeks**<br>p=0.2013<br>**C9ORF72-GR400-B6J:WT vs C9ORF72-GR400-B6J:Het at 70 weeks**<br>p=0.1653<br><br>**SPR (based on frequency) – compare genotype at every age**<br>**C9ORF72-GR400-B6J:WT vs C9ORF72-GR400-B6J:Het at 18.5 weeks**<br>p=0.2845<br>**C9ORF72-GR400-B6J:WT vs C9ORF72-GR400-B6J:Het at 70 weeks**<br>p=0.3665<br><br>**Distance travelled – habituation – compare age at every genotype**<br>**18.5 vs 70 weeks, C9ORF72-GR400-B6J:WT**<br>p=0.3296<br><br>**18.5 vs 70 weeks, C9ORF72-GR400-B6J:Het**<br>p=0.0302

Disease Models & Mechanisms • Supplementary information

	**Distance travelled – test**<br>dist_test_overall~genotype*(mean_experimental_age + sex + inh) + (1|animal_name)	**Distance travelled – test**<br>**mean_experimental_age** F(1, 38.859)=9.2378, p=0.004227 **	**Distance travelled – test – compare age at every genotype**<br>**18.5 vs 70 weeks, C9ORF72-GR400-B6J:WT**<br>p=0.3672<br><br>**18.5 vs 70 weeks, C9ORF72-GR400-B6J:Het**<br>p=0.0110
**Sanderson Y-maze forced alteration**	**Distance travelled - habituation**<br>dist_hab_overall~genotype*(mean_experimental_age + sex + inh) + (1|animal_name)<br><br>**Distance travelled - test**<br>dist_test_overall~genotype*(mean_experimental_age + sex + inh) + (1|animal_name)<br><br>**Novel preference ratio (NPR, based on time)**<br>NPRtime~genotype*(mean_experimental_age + sex + inh) + (1|animal_name)<br><br>**NPR (based on frequency)**<br>NPRfreq~genotype*(mean_experimental_age + sex + inh) + (1|animal_name)	**Distance travelled - habituation**<br>**mean_experimental_age** F(1, 40.516)= 4.8324, p=0.0337 *<br><br>**Distance travelled - test**<br>**mean_experimental_age** F(1, 42.926)=5.3118, p=0.02608 *<br><br>**NPR (based on time)**<br>**genotype:mean_experimental**_F(1, 40.784)= 5.4534, p=0.02453 *<br><br>**NPR (based on frequency)**<br>**genotype:mean_experimental_age** (F1, 41.552)=5.6421, p=0.02223 *<br>**sex** F(1, 41.318)=5.7870, p=0.02070 *	**Distance travelled – habituation – compare age at every genotype**<br>**12.5 vs 64.5 in C9ORF72-GR400-B6J:WT**<br>p=0.0596<br><br>**12.5 vs 64.5 in C9ORF72-GR400-B6J:Het**<br>p=0.7970<br><br>**Distance travelled – test – compare age at every genotype**<br>**12.5 vs 64.5 in C9ORF72-GR400-B6J:WT**<br>p=1.0000<br><br>**12.5 vs 64.5 in C9ORF72-GR400-B6J:Het**<br>p=0.0159<br><br>**NPR (based on time) – compare genotype at every age**<br>**C9ORF72-GR400-B6J:WT vs C9ORF72-GR400-B6J:Het at 12.5 weeks**<br>p=1.0000<br><br>**C9ORF72-GR400-B6J:WT vs C9ORF72-GR400-B6J:Het at 64.5 weeks**<br>p=0.0828<br><br>**NPR (based on frequency) – compare genotype at every age**<br>**C9ORF72-GR400-B6J:WT vs C9ORF72-GR400-B6J:Het at 12.5 weeks**<br>p=1.0000<br><br>**C9ORF72-GR400-B6J:WT vs C9ORF72-GR400-B6J:Het at 64.5 weeks**<br>p=0.0308<br><br>**Distance travelled – test – compare sex at every genotype**<br><br>**Male vs Female, C9ORF72-GR400-B6J:WT**<br>p=0.1894<br><br>**Male vs Female, C9ORF72-GR400-B6J:Het**<br>p=0.2016

Tardbp$^{Q331K/Q331K}$ study

Behavioural test	Statistical model	Significant main effects (F statistics)	Significant post hoc effects
**Weight**	averageweight~genotype*(age_weeks + sex) + age_weeks*sex + (1|animal_name)	**genotype** F(1, 50.30)=16.1582, p=0.0001952 ***<br>**age_weeks** F(41, 1674.58)=313.2932 p< 2.2e-16 ***<br>**sex** F(1, 50.29)= 36.5988 p=1.796e-07 ***<br>**genotype:age_weeks** F(41, 1674.60)=16.2455 p< 2.2e-16 ***<br>**age_weeks:sex** F(41, 1674.59)= 3.3519, p=9.666e-12 ***	**TDP-43_Q331K:WT vs TDP-43_Q331K:Hom females** - significant difference from 48 weeks, p= 0.0141. Stays significant for all ages onwards, at 72 weeks - p<0.0001<br>**TDP-43_Q331K:WT vs TDP-43_Q331K:Hom males** - significant difference from 54 weeks, p=0.0199. Stays significant for all ages onwards apart from 56 weeks of age, , at 72 weeks - p<0.0001
**Body composition (echo MRI)**	**Fat mass**<br>fatmass~genotype*(mean_experimental_age + sex) + mean_experimental_age*sex +(1|animal_name)	**Fat mass**<br>**genotype** F(1, 52.194)=23.8519, p=1.026e-05 ***<br>**mean_experimental_age** F(4, 179.274)=185.2816 p< 2.2e-16 ***<br>**sex** F(1, 52.391)=9.0546 p=0.004022 **<br>**genotype:mean_experimental_age** F(4, 179.296)=15.4586 p=6.982e-11 ***	**Fat mass**<br>**TDP-43_Q331K:WT vsTDP-43_Q331K:Hom, female**<br>p=0.000212 – from 48 weeks<br><br>**TDP-43_Q331K:WT vsTDP-43_Q331K:Hom, male**<br>p=0.018 – from 64 weeks

<table>
<tr><td></td><td>Lean mass
leanmass~genotype*(mean_experimental_age + sex) + mean_experimental_age*sex +(1|animal_name)</td><td>genotype:sex F(1,51.913)=5.3641 p=0.024539 *
mean_experimental_age:sex F(4, 179.295)=3.7686 p=0.005721 **

Lean mass
genotype F(1 49.137)=11.6177, p= 0.001313 **
mean_experimental_age F(4, 178.301)= 73.0793 p< 2.2e-16 ***
sex F(1, 49.319)=602.5097 p< 2.2e-16 ***
genotype:mean_experimental_age F(4, 178.374)=2.9340, p=0.022142 *</td><td>Lean mass
TDP-43_Q331K:WT vsTDP-43_Q331K:Hom, male
p=0.0046 - from 64 weeks;</td></tr>
<tr><td>Olfaction test</td><td colspan="3">Table 3 C and D</td></tr>
<tr><td>Optokinetic drum (OKD)</td><td>acuity~genotype*(mean_experimental_age+sex) +(1|animal_name)</td><td>mean_experimental_age F(1, 48.841)=26.9056, p=4.09e-06 ***</td><td>Compare age at every genotype
16 vs 68 weeks TDP-43_Q331K:WT
p=0.0012

16 vs 68 weeks TDP-43_Q331K:Hom
p=0.0014</td></tr>
<tr><td>Elevated plus maze (EPM)</td><td>Duration
Duration~genotype*(sex + Section) + (1|animal_name)

Frequency
Frequency~genotype*(sex + Section) + (1|animal_name)</td><td>Duration
Section F(2, 151)=438.1583, p< 2e-16 ***

Frequency
Section F(2, 102)=598.5292, p< 2e-16 ***

genotype:Section F(2, 102)=4.6999, p=0.01116 *</td><td>Duration
No effects of interest to explore

Frequency – compare genotype at every section
TDP-43_Q331K:WT vs TDP-43_Q331K:Hom closed section p=0.0723

TDP-43_Q331K:WT vs TDP-43_Q331K:Hom open section p=1.0000

TDP-43_Q331K:WT vs TDP-43_Q331K:Hom centre
p=0.2389</td></tr>
<tr><td>Marble burying</td><td>kruskal.test
(marbles_burried ~ genotype)</td><td>14 weeks
Kruskal-Wallis chi-squared = 0.24674, df = 1, p-value = 0.6194

67 weeks
Kruskal-Wallis chi-squared = 4.2548, df = 1, p-value = 0.03914*</td><td>NA</td></tr>
<tr><td>Crawley three-chamber social preference test</td><td>Time spent with mouse
time_m~genotype*(mean_experimental_age+sex) + (1|animal_name)

Time spent with object
time_object~genotype*(mean_experimental_age +sex)+ (1|animal_name)

SPR (based on time)
SPRtime~genotype*(mean_experimental_age + sex) + (1|animal_name)

SPR (based on frequency)
SPRfrequency~genotype*(mean_experimental_age + sex) + (1|animal_name)

Distance travelled - habituation
dist_hab_overall~genotype*(mean_experimental_age + sex) + (1|animal_name)

Distance travelled – test
dist_test_overall~genotype*(mean_experimental_age + sex) + (1|animal_name)</td><td>Time spent with mouse
mean_experimental_age F(1, 88)=6.3402, p=0.01361 *

Time spent with object
mean_experimental_age F(1, 88)= 4.5926, p=0.03487 *

SPR (based on time)
No significant main effects

SPR (based on frequency)
No significant main effects

Distance travelled - habituation
mean_experimental_age F(1, 47.656)=29.8108, p=1.688e-06 ***

genotype:mean_experimental_age F(1, 47.656)= 5.6598, p=0.02141 *

Distance travelled – test
genotype F(1,46.964)=15.1361, p=0.0003141 ***
mean_experimental_age F(1 42.066)=29.2676, p=2.776e-06 ***
sex F(1, 46.453)=4.5465, p=0.0383032 *
genotype:mean_experimental_age F(1, 42.066)=9.2153, p=0.0041070 **</td><td>Time spent with mouse – compare age at every genotype
18 vs 71 weeks TDP-43_Q331K:WT
p=0.3249

18 vs 71 weeks TDP-43_Q331K:Hom
p=0.0773

Time spent with object – compare age at every genotype
18 vs 71 weeks TDP-43_Q331K:WT
p=0.1977

18 vs 71 weeks TDP-43_Q331K:Hom
p=0.3757

SPR (based on time)
NA

SPR (based on frequency)
NA

Distance travelled – habituation – compare genotype at every age
TDP-43_Q331K:WT vs TDP-43_Q331K:Hom, 18 weeks
p=1.0000

TDP-43_Q331K:WT vs TDP-43_Q331K:Hom, 71 weeks
p=0.0162

Distance travelled – test – compare genotype at every age
TDP-43_Q331K:WT vs TDP-43_Q331K:Hom, 18 weeks
p=0.2505

TDP-43_Q331K:WT vs TDP-43_Q331K:Hom, 71 weeks
p=<.0001

Distance travelled – test – compare sex at every genotype

Male vs Female, TDP-43_Q331K:WT
p=0.6269

Male vs Female, TDP-43_Q331K:Hom
p=0.1041</td></tr>
</table>

Disease Models & Mechanisms • Supplementary information

**Sanderson Y-maze forced alteration**	**<u>Distance travelled - habituation</u>**<br>dist_hab_overall~genotype*(mean_experimental_age + sex) + (1|animal_name)<br><br>**<u>Distance travelled - test</u>**<br>dist_test_overall~genotype*(mean_experimental_age + sex) + (1|animal_name)<br><br>**<u>NPR (based on time)</u>**<br>NPRtime~genotype*(mean_experimental_age + sex) + (1|animal_name)<br><br>**<u>NPR (based on frequency)</u>**<br>NPRfreq~genotype*(mean_experimental_age + sex) + (1|animal_name)	**<u>Distance travelled - habituation</u>**<br>**genotype** F(1, 47.779)= 6.6680, p=0.01294 *<br><br>**mean_experimental_age** F(1, 43.624)=50.8120, p=7.768e-09 ***<br><br>**genotype:mean_experimental_age** F(1, 43.624)=4.8058, p=0.03375 *<br><br>**<u>Distance travelled - test</u>**<br>**sex** F(1, 46.949)= 8.6875, p=0.004977 **<br>**genotype:mean_experimental_age** F(1, 43.114)=4.4811, p=0.040082 *<br><br>**<u>NPR (based on time)</u>**<br>**mean_experimental_age** F(1, 44.249)=4.3377, p=0.04309 *<br><br>**<u>NPR (based on frequency)</u>**<br>**mean_experimental_age** F(1 41.727)=21.5518, p=3.408e-05 ***	**<u>Distance travelled – habituation – compare genotype at every age</u>**<br>**TDP-43_Q331K:WT vs TDP-43_Q331K:Hom, 12.5 weeks** p = 0.7372<br><br>**TDP-43_Q331K:WT vs TDP-43_Q331K:Hom, 65.5 weeks** p = 0.0041<br><br>**<u>Distance travelled – test – compare genotype at every age</u>**<br>**TDP-43_Q331K:WT vs TDP-43_Q331K:Hom, 12.5 weeks** p = 1<br><br>**TDP-43_Q331K:WT vs TDP-43_Q331K:Hom, 65.5 weeks** p = 0.0356<br><br>**<u>Distance travelled – test – compare sex at every genotype</u>**<br><br>**Male vs Female, TDP-43_Q331K:WT**<br>p=0.0522<br><br>**Male vs Female, TDP-43_Q331K:Hom**<br>p=0.1360<br><br>**<u>NPR (based on time) – compare age at every genotype</u>**<br>**12.5 vs 65.5 weeks, TDP-43_Q331K:WT** p = 0.0174<br><br>**12.5 vs 65.5 weeks, TDP-43_Q331K:Hom** p = 1.0000<br><br>**<u>NPR (based on frequency) – compare age at every genotype</u>**<br>**12.5 vs 65.5 weeks, TDP-43_Q331K:WT** p = 0.0005<br>**12.5 vs 65.5 weeks, TDP-43_Q331K:Hom** p = 0.0315
